# Two-way photoswitching norbornadiene derivatives for solar energy storage†

**DOI:** 10.1039/d4sc04247f

**Published:** 2024-09-26

**Authors:** Liang Fei, Helen Hölzel, Zhihang Wang, Andreas Erbs Hillers-Bendtsen, Adil S. Aslam, Monika Shamsabadi, Jialing Tan, Kurt V. Mikkelsen, Chaoxia Wang, Kasper Moth-Poulsen

**Affiliations:** a College of Textile Science and Engineering, Jiangnan University 1800 Lihu Road 214122 Wuxi China wangchaoxia@sohu.com; b Department of Chemical Engineering, Universitat Politècnica de Catalunya, EEBE Eduard Maristany 10-14 08019 Barcelona Spain kasper.moth-poulsen@upc.edu; c School of Engineering, College of Science and Engineering, University of Derby Markeaton Street Derby DE22 3AW UK; d Department of Chemistry, University of Copenhagen Universitetsparken 5 2100 Copenhagen Denmark; e Department of Chemistry and Chemical Engineering, Chalmers University of Technology Gothenburg 41296 Sweden; f The Institute of Materials Science of Barcelona, ICMAB-CSIC Bellaterra 08193 Barcelona Spain; g Catalan Institution for Research & Advanced Studies, ICREA Pg. Lluís Companys 23 Barcelona Spain

## Abstract

Molecular photoswitches of norbornadiene (NBD) derivatives have been effectively applied in molecular solar-thermal energy storage (MOST) by photoisomerization of NBD to a quadricyclane (QC) state. However, a challenge of the NBD-based MOST system is the lack of a reversible two-way photoswitching process, limiting conversion from QC to thermal and catalytic methods. Here we design a series of NBD derivatives with a combination of acceptor and donor units to achieve two-way photoswitching, which can optically release energy by back-conversion from QC to NBD. Highly efficient photoconversion yields from NBD to QC and QC to NBD are up to 99% and 82%, respectively. The energy storage density of two-way photoswitching NBD is up to 312 J g^−1^ and optically controlled two-way photoswitching devices are demonstrated for the first time both in flow and in thin films, which illustrate a promising approach for fast and robust energy release in both solution and solid state.

## Abstract 2

A series of two-way photoswitching norbornadiene derivatives is designed for efficient and robust energy conversion. A flow MOST device and MOST films demonstrate optically controlled energy release in solution and solid state, respectively.

## Graphical abstract

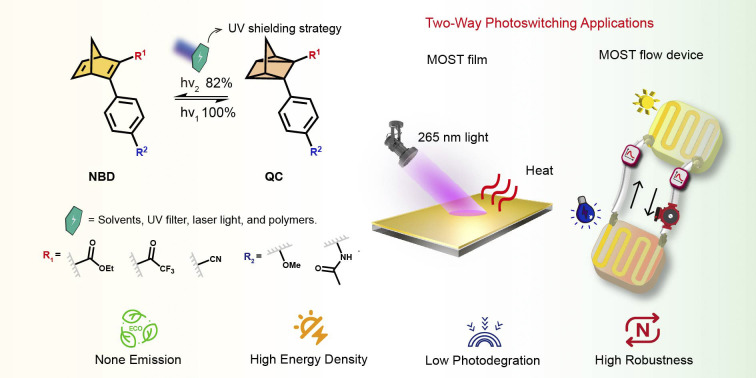

## Introduction

Fossil fuels currently dominate global energy consumption and may continue to increase during the next 30 years, exacerbating the already serious environmental impacts.^1,2^ To address this issue, molecular solar thermal energy storage (MOST) systems are being developed as a possible emission-free energy storage concept.^3–7^ Unlike combustible fuels, MOST materials can directly convert solar energy into chemical energy through a photoisomerization reaction.^8–13^ Among the most promising MOST materials are derivatives of norbornadiene–quadricyclane (NBD–QC), known for their high energy storage density and long-term energy storage capabilities.^14–18^

The stored energy can be released on demand, occurring either spontaneously or through external stimuli, such as electrocatalytic, catalytic, and light.^19–26^ While both electrocatalytic and catalytic approaches have been developed to trigger energy-releasing conversion from QC to NBD,^21,23^ it's worth noting that the catalytic system requires physical colocation in fixed bed reactors with the MOST system to operate. To date, very few research activities have been focusing on NBD-based MOST devices in the solid state,^27,28^ due to the inefficient energy release method. In contrast to other photoswitching systems, such as azobenzenes, which can be reversibly switched between *Z* and *E* forms with different wavelengths of light,^29–31^ there are very few examples of two-way photoswitching NBD–QC systems,^32–34^ and until now, none of them have been demonstrated to work in the solid state.

To this point, photoisomerization from QC to NBD has been limited due to low photoisomerization yields and the need for short ultraviolet (UV) irradiation wavelengths to activate the QC system. In the case of unsubstituted NBD, it exhibits no absorption beyond 210 nm, necessitating deep UV irradiation for QC to NBD conversion.^35^ To bathochromically shift absorbing wavelengths, resulting in a better overlap with the solar spectrum, donor–acceptor groups have been introduced into NBD.^36–39^ Additionally, the photodegradation of NBD, particularly under short UV light, has restricted the development of light-triggered systems.^40,41^ Guldi *et al.* reported a photodegradation mechanism for NBD with ester groups, wherein short UV light (258 nm) led to a localized excited state that formed a bicyclo[4.1.0] derivative,^42^ rendering the formed products unresponsive to photoisomerization. Therefore, addressing these challenges is essential for the development of an efficient optical energy release approach, both in solution and the solid state.

Herein, we introduce a series of NBD-based molecules specifically designed for efficient two-way photoswitching. This was achieved by incorporating acceptor groups such as ester, trifluoroacetyl, and cyano, paired with carefully chosen donors, including benzene substituted with methoxy or amide groups. To mitigate photodegradation, we have employed a tunable UV shielding strategy based on solvents or the polymer matrix to cut-off short UV light, thus enhancing the cyclability of two-way photoswitching. Furthermore, to illustrate the functionality of two-way photoswitching, we have, for the first time, established a continuously operating photo-triggered liquid flow MOST device and demonstrated solid MOST films capable of two-way photoswitching. These findings provide promising pathways for implementing two-way photoswitching NBD-based MOST systems, both in solution and in the solid state.

## Introduction

### Two-way photoswitching properties

**Fig. 1:**
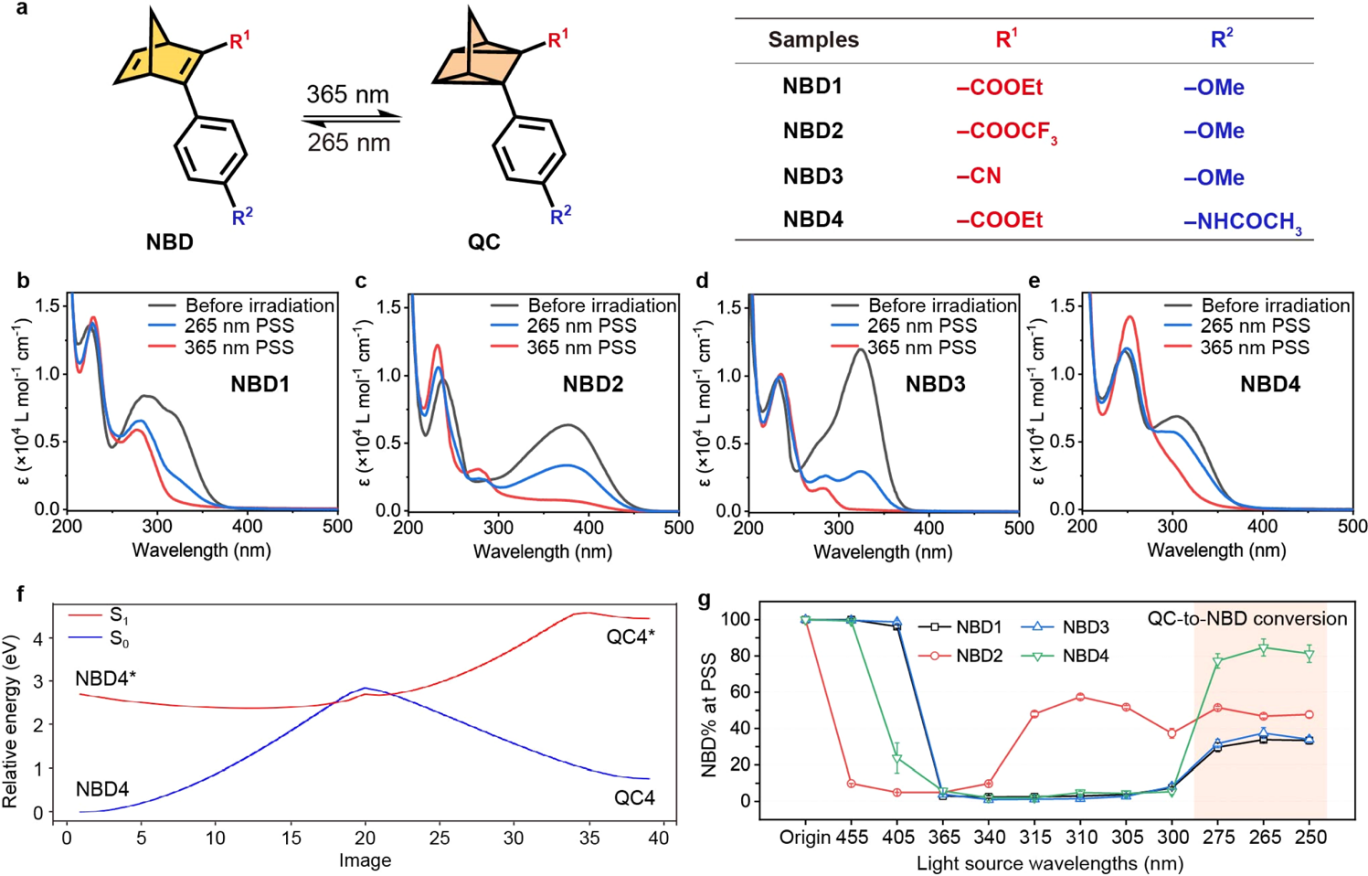
Molecular structure and two-way photoswitching properties of designed NBDs. (a) Molecular structure of two-way photoswitching NBDs. UV-Vis absorption spectra of (b) NBD1, (c) NBD2, (d) NBD3, and (e) NBD4 in an MeCN solution (0.1 mM L^−1^). (f) Photoisomerization paths between NBD4 and QC4 *via* the minimum energy conical intersection. (g) Photoisomerization yields using 11 different irradiation wavelengths of NBDs at photostationary states (PSSs) in a MeCN solution.

To identify NBD–QC derivatives that can efficiently serve as two-way photoswitches, we designed four NBD-based molecules incorporating a donor–acceptor system (Fig. 1a). The optical properties of NBDs exhibited a photostationary state (PSS) under both 365 nm and 265 nm light irradiation. For example, NBD4 exhibited two broad absorption peaks with high maximum absorptivities (*λ*_max_) at 312 nm and 248 nm, respectively (Fig. 1b and S1†). The onset wavelength (*λ*_onset_) of NBD4 extended to 395 nm, enabling it to potentially absorb 5.4% of the entire solar spectrum (Fig. S3†). During 365 nm light irradiation, NBD → QC isomerization was observed in NBD4, with the absorption peak at *λ*_max_ = 312 nm disappearing and a stronger absorption peak emerging at 251 nm. The ratios of NBD or QC (NBD% or QC%), at the original state and 365 nm PSS, were calculated by ^1^H NMR (Fig. S5–S9†). The isomerization conversion yields (NBD → QC) for all NBDs were above 95%, indicating efficient NBD → QC isomerization.

Efficient isomerization from QC to NBD is essential for a functioning MOST system, with external stimuli facilitating this isomerization for device design. Short UV light irradiation (*e.g.*, 265 nm light) into QC-centered absorption (*e.g.*, 251 nm for NBD4) activated QC to NBD isomerization in all designed NBDs (Fig. 1b–e). The optical back conversion yields were in a range of 31% to 82% (Table 1), attributable to the overlap of the *σ* → σ* transition in NBD and QC. One of the most intricate challenges of two-way photoswitching is optical overlap between two isomers, and also for the NBD–QC system presented here. Notably, the optical back-conversion from QC4 to NBD4 achieved a high yield of 82% under 265 nm light irradiation (Table S1†). The back-isomerization under 265 nm light adhered to first-order reaction kinetics (*k* = 0.190 min^−1^), similar to NBD to QC conversion (Fig. S4†). The reaction coordinates going from NBD to QC between S_1_ and S_0_ were calculated (left to right in Fig. 1f and S2†), which fit the low-lying intersection model between the photochemically relevant excited state and the ground state.^43,44^ This indicates that all designed NBDs could feature a two-way photoswitching function, and the excited energy from QC to NBD is higher than that of NBD-to-QC conversion.

**Table 1:** Two-way photoswitching parameters of NBDs

Samples	*λ*_onset_ a (nm)	QY (%)		*t*_1/2_ at 25 °C (days)	Photoconversion yield (NBD%)	
		NBD to QC	QC to NBD		At 365 nm	At 265 nm
NBD1	385	62	26	317	97	31
NBD2	460	28	—b	0.002	—b	—b
NBD3	381	37	39	48	99	38
NBD4	395	75	53	9772	99	82

a Absorption onset is defined as log *ε* = 2.

b The thermoback conversion of NBD2 is too fast to test the QY and photoconversion yields under 265 nm irradiation.

The optical back-conversion yields at the PSS are highly dependent on both the molecular structure and on the wavelengths of light irradiation used. Under specific wavelength irradiation, both photoisomerization processes took place in spectral overlap regions of both isomers, until reaching the dynamic equilibrium of isomers. At 365 nm irradiation, the percentages of NBD (NBD%) decreased for all NBDs (Fig. 1g), due to near quantitative conversion to QC. Even under 455 nm light irradiation, NBD2 to QC2 conversion was observed, owing to the high onset of absorption. The *λ*_onset_ of NBD1 was 385 nm, thus allowing for isomerization to occur under 365 nm light. Irradiating QC with shorter wavelengths increased the conversion to NBD due to increased competing optical absorption between QC and NBD. Furthermore, 265 nm light irradiation resulted in the highest conversion to NBD, indicating efficient energy release.

The photoisomerization quantum yields (*Φ*) for NBD to QC and QC to NBD were determined in acetonitrile solution to investigate the efficiency of the photoisomerization event. The *Φ* of NBD4 under 365 nm irradiation was 75%, indicating that most of the absorbed photons successfully facilitated NBD to QC photoisomerization (Tables 1 and S2–S10†). Meanwhile, the *Φ* of back conversion (QC to NBD, 53%) was slightly lower than that of forward isomerization.

Maximizing the duration of energy storage for long-term two-way photoswitching is crucial. The thermal back-conversion rates of all NBDs were examined at various temperatures (Fig. S10–S13†) and the half-life (*t*_1/2_) values at room temperature calculated by Eyring analysis (Tables 1, S11, Fig. S14 and S15†). These *t*_1/2_ values correlated strongly with the absorption spectrum (*λ*_onset_),^15,37,45^ and the energy storage time is shortened with the redshifted absorption of donor–acceptor molecules. NBD2 has a *t*_1/2_ value of only 0.002 days (172 s), leading to an unusual fluctuation of isomer ratios in the range of 340 nm to 300 nm (Fig. 1g). The back-conversion *t*_1/2_ values of NBD1, NBD3, and NBD4 reached 317, 48, and 9772 days in acetonitrile solution, respectively, signifying significant long-term stability. For example, the energy stored during the summer by NBD4 can retain at least 90% of its original capacity when utilized in the winter, ensuring minimal loss over seasonal storage periods.

### Photostability and photodegradation

**Fig. 2:**
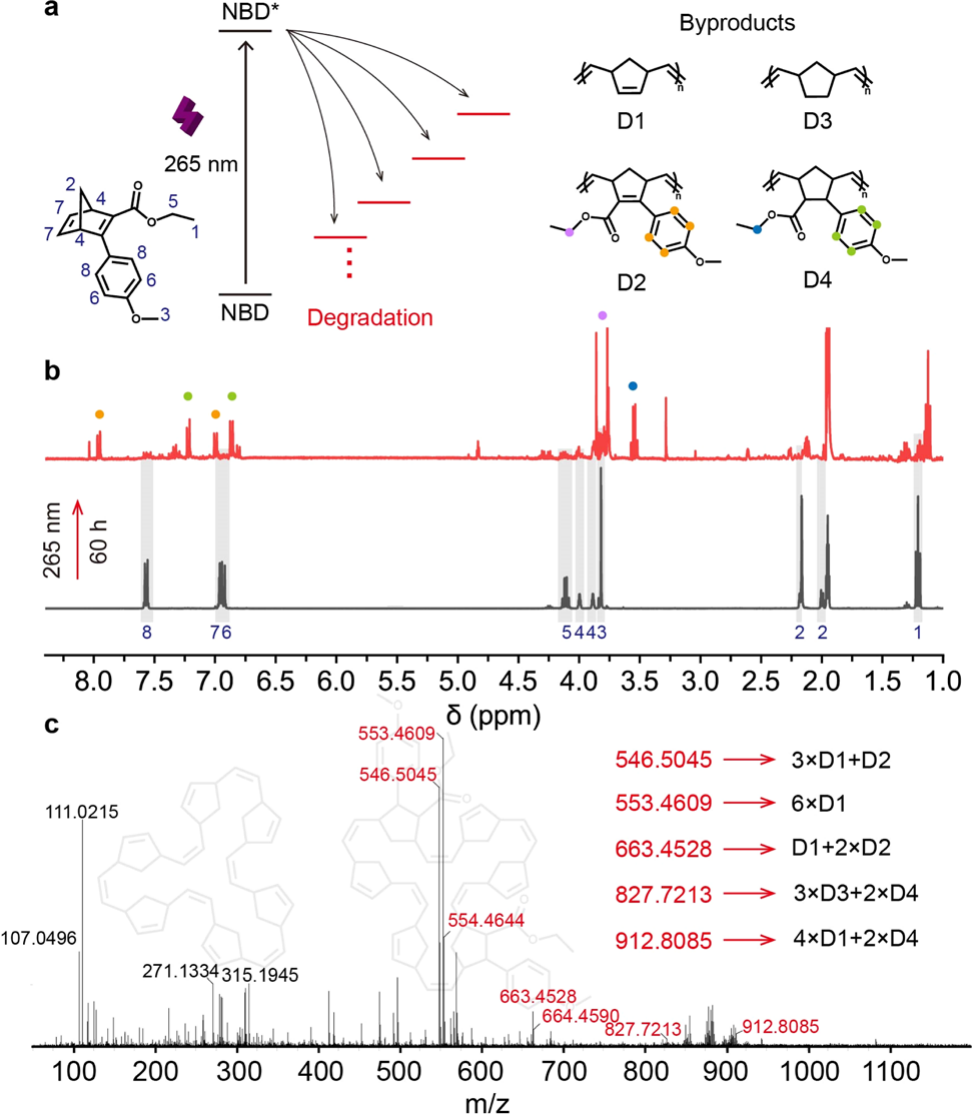
Photodegradation and its byproducts of NBD1. (a) Photodegradation mechanism NBD1 under 265 nm light irradiation. (b) ^1^H NMR spectra and of NBD1 before and after 265 nm light irradiation. (c) HRMS spectrum of NBD1 after 265 nm light irradiation.

Although all designed NBDs exhibit successful two-way photoswitching with high conversion yields, photodegradation remains a challenge that in some cases limits their potential application. In contrast to other photoswitches such as azobenzene and stilbene,^46^ the bond formation and breaking involved in the isomerization between NBD and QC pose a higher risk of forming byproducts.^3,40,47,48^ Predicting the products and yields of photoreactions during the photoreaction is more complex than that during thermally induced reactions.^49^ Thus, we investigated the byproducts of NBD1 (NBD1-d) under 265 nm light irradiation (Fig. 2a), using ^1^H NMR and mass spectrometry (Fig. 2b and c), concluding that NBD molecules might undergo ring-opening metathesis copolymerization (ROMP)^50,51^ under extended exposure to light.

For a better understanding of the photoisomerization in the NBD–QC system, especially under short UV light irradiation, we recorded ^1^H NMR spectra (Fig. 2b, and S16–S18†). Protons on the phenyl ring of NBD1 and polymers by ROMP (D2 and D4) served as spectral markers to monitor photodegradation. In comparison to the ^1^H NMR spectrum of pure NBD1, a multitude of new peaks emerged in the degradation spectrum that was recorded after up to 60 hours of 265 nm light irradiation. All original peaks from pure NBD1 nearly vanished after light irradiation, revealing significant photodegradation. Proton shifts at 7.58 and 6.92 ppm in NBD1 converted to 7.93 and 7.01 ppm for D2, and 7.47 and 6.78 ppm for D4. A comparison of this degradation sample with the original NBD1 indicated that compounds with larger molecular weights were formed under 265 nm light irradiation, consistent with the calculated structures. For example, a byproduct with a molecular weight of 546.5045 g mol^−1^ can be polymerized by three D1 monomers and one D2 monomer (Fig. 2c, S21 and S22†). Thus, 265 nm light facilitates QC to NBD conversion, inevitably leading to ROMP.

**Fig. 3:**
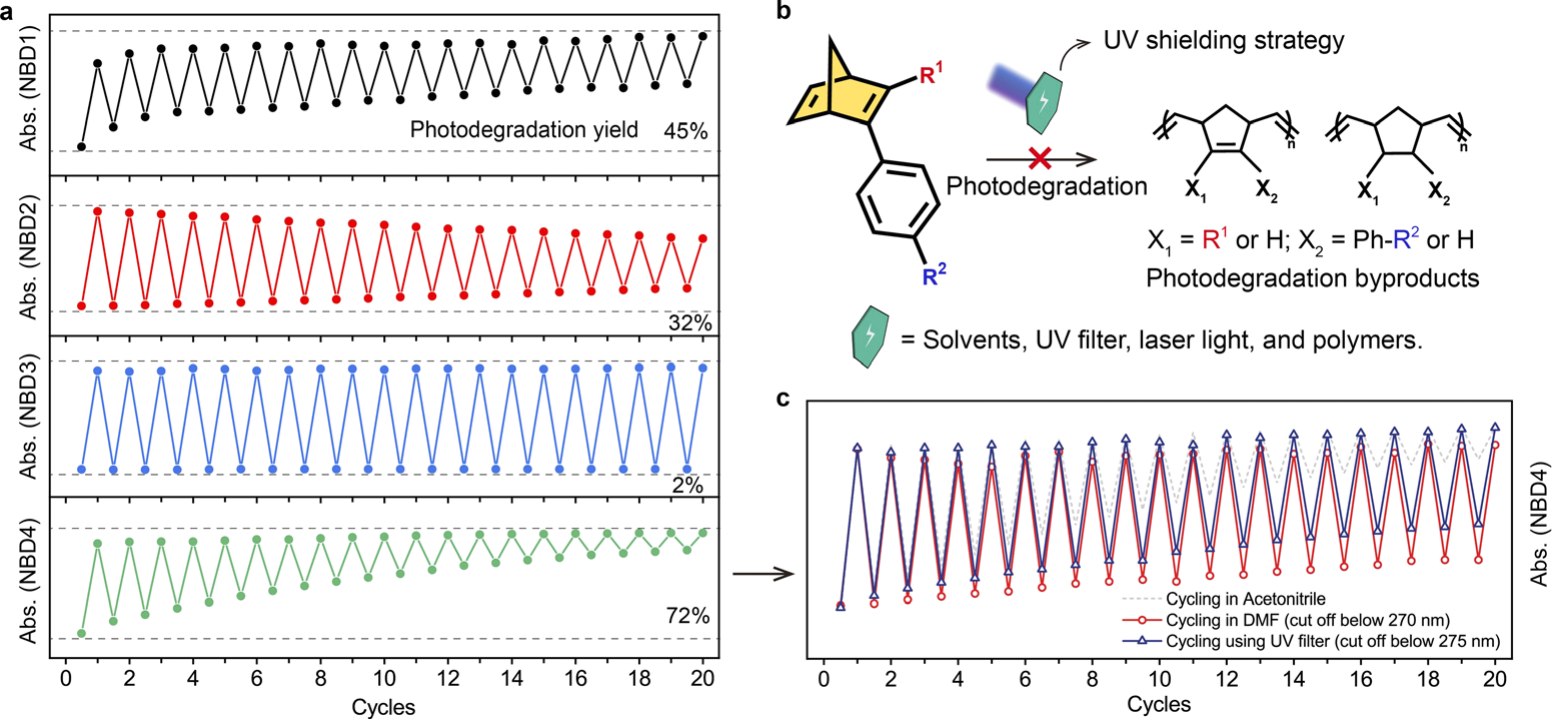
Photostability of NBDs under two-way photoswitching cycling. (a) Two-way photoswitching cycling test and photodegradation yields of NBDs in MeCN (0.1 mM L^−1^), where NBD to QC conversion and subsequent QC to NBD back-conversion occur by 365 nm and 265 nm light irradiation, respectively. (b) Scheme of photostability improvement by UV shielding protection. (c) Two-way photoswitching cycling test of NBD4 using solvent and a light filter to cut off deep UV wavelengths.

To evaluate photostability further, we subjected all NBDs to a two-way photoswitching cycling test. After undergoing 20 cycles, NBD3 with a cyano group exhibited excellent robustness, with only 2% degradation (Fig. 3a). However, the photodegradation yields of NBD1 and NBD4, both with ester groups, reached 45% and 72%, limiting their rechargeability during solar energy conversion. We also compared the photodegradation yields during optical back-conversion (265 nm light) to those *via* thermal back-conversion (Fig. S23†). The latter exhibited less degradation, illustrating that short UV light irradiation leads to more severe degradation. The UV-Vis absorption curves of NBDs shifted to lower wavelengths relative to those of original NBDs, while the absorption onset wavelengths extended to higher wavelengths (Fig. S24†). NBDs-d lost the ability for photoisomerization after prolonged 265 nm light irradiation, indicating their inability to store and convert solar energy.

Considering the occurrence of photodegradation induced by short UV light irradiation, we explored a tunable UV shielding strategy to enhance the photostability of NBD. This UV shield can eliminate short UV light while retaining monochromatic light for QC to NBD conversion (Fig. 3b). A solvent, UV filter, polymer, or other means can serve as the UV shield. Using solvents with cutoff wavelengths, such as *N*,*N*-dimethylformamide (DMF), can effectively block input light below 270 nm. Notably, the photodegradation yield of NBD4 significantly decreased after 20 cycles of alternate light irradiation from 72%, to just 22%, revealing that the UV shield can substantially improve photostability (Fig. 3c). A commercial UV filter, which cuts off light wavelengths below 275 nm, yielded a similar result. Therefore, this tunable UV shielding strategy efficiently enhances photostability without sacrificing the optical back-conversion function, making it a promising approach for all NBD molecules.

### Solar energy storage and release using light

**Fig. 4:**
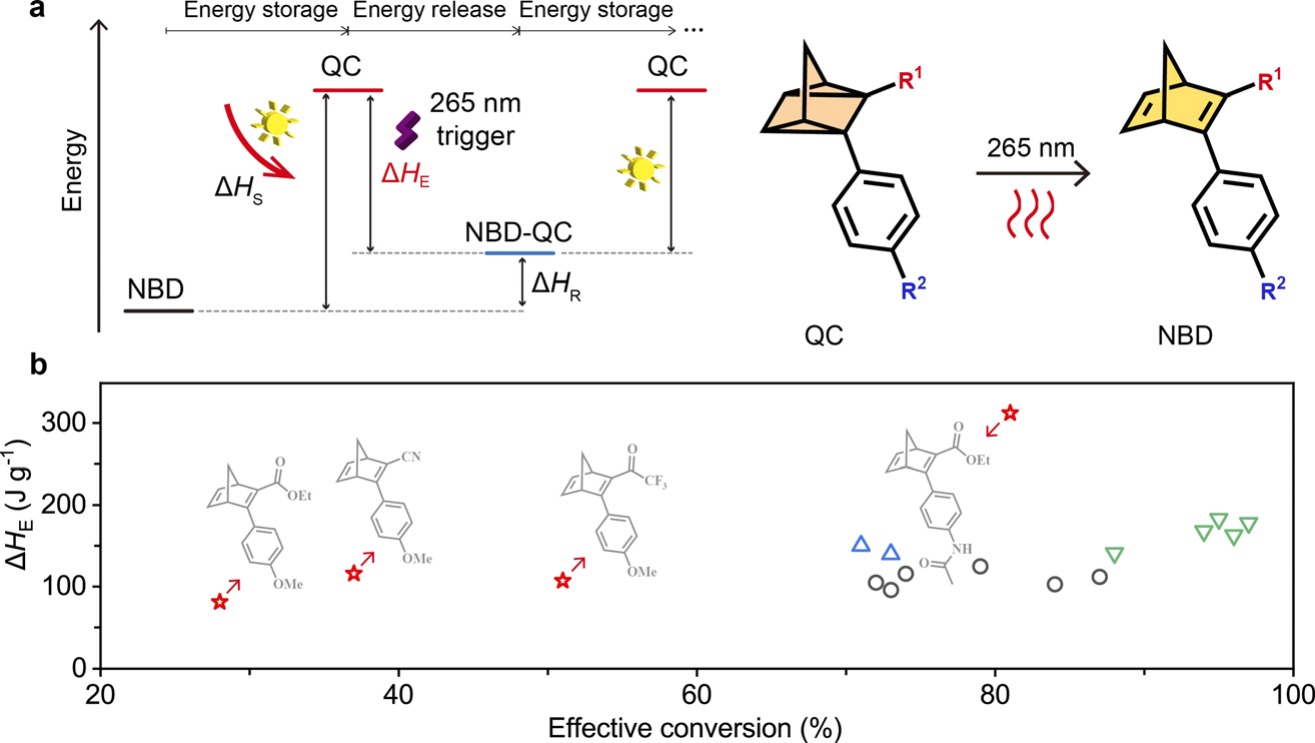
Energy storage and release under two-way photoswitching cycles. (a) Illustration of a two-way photoswitching MOST system. (b) A comparison of the energy density from the latest reports in two-way photoswitching molecules: ○, alkyl azobenzene derivatives;^52–56^ △, *ortho*-functionalized azobenzene derivatives;^57^ ▽, pyrazole-based azobenzene derivatives;^6,9,58^ ☆, and this paper.

The energy storage capacity of the two-way photoswitching NBD–QC system depends on the isomerization energy of QC to NBD conversion. The stored energy enthalpy (Δ*H*_S_) was measured after the charging process (365 nm irradiation) and the residual energy enthalpy (Δ*H*_R_) after the discharging process (265 nm irradiation) (Fig. 4). The effective energy storage enthalpy (Δ*H*_E_) is calculated using Δ*H*_S_ − Δ*H*_R_ (Fig. 4a). Exothermic peaks were observed by means of differential scanning calorimetry for all NBDs during the heating process at a rate of 5 °C min^−1^, and the integrated area of these peaks represented the isomerization energy enthalpy (Fig. 4b).

The photoconversion yields of QC to NBD can significantly affect how efficient energy storage capacity will be in practice. NBD4 possessed the lowest Δ*H*_R_ among all NBDs and the Δ*H*_E_ reaching 312 J g^−1^ (Fig. S25 and Table S12†). The Δ*H*_E_ of NBD4 by optical back-conversion is superior to that of previously reported two-way photoswitching MOST materials that we could find in the literature (Fig. 4b).^6,9,52–58^ This illustrates that energy storage of NBD–QC derivatives using a two-way photoswitching strategy offers a promising avenue for applications in the solution and, notably, in the solid state.

### MOST flow device

**Fig. 5:**
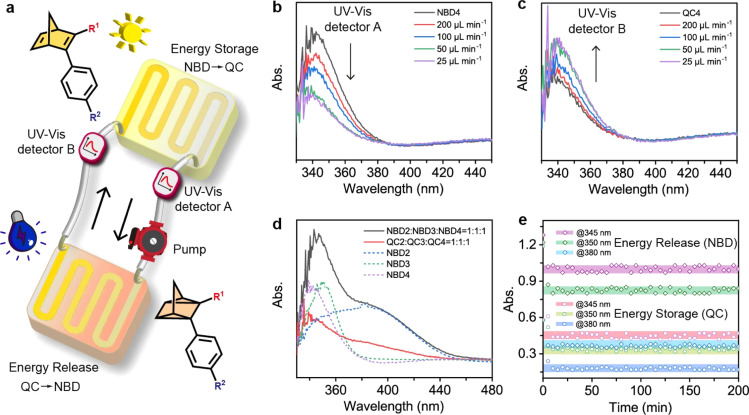
Two-way photo-switching system in an automated flow device. (a) Automated flow device incorporating the two-way photoswitching MOST system. UV-Vis detectors A and B tested the photoisomerization states after 365 nm and 265 nm light irradiation, respectively. UV-Vis absorption spectra of NBD4 with flow rates in (b) UV-Vis detector A and (c) detector B. (d) UV-Vis absorption spectra of the multi-NBD system. (e) Dynamic photoisomerization processes in the automation flow MOST device.

MOST flow devices have been applied in several demonstrations of light harvesting and heat utilization.^22,23,59–61^ The reported flow MOST devices with NBD–QC derivatives have until now been based on one-way photoswitching, where energy storage was achieved using light and energy release was triggered by catalysts or heat.^22,23,27,36,37,40^ Capitalizing on efficient optical QC to NBD conversion, we here constructed a two-way photoswitching microfluidic reactor setup (Fig. 5a and S26†). For the close-cycled flow MOST device, all NBD solutions passed through a solar energy capture reactor (62.5 μL) and were completely converted to the QC state (Movie S1†), with the QC solution being back-converted to the NBD state by 265 nm irradiation. Moreover, the flow rate was optimized for maximum energy utilization efficiency. With decreasing flow rates from 200 to 50 μL min^−1^, the absorption in UV-Vis detector A at 350 nm decreased (Fig. 5b), which indicated that the NBD4 solution was irradiated for a longer time to facilitate the NBD4 to QC4 conversion. At a lower flow rate (25 μL min^−1^), the absorption curve nearly overlapped with that at a flow rate of 50 μL min^−1^, due to complete forward conversion. The flow-rate-dependent absorption in UV-Vis detector B is similar to that in UV-Vis detector A (Fig. 5c), illustrating that QC to NBD conversion under 265 nm light reached a photostationary state at a flow rate of 50 μL min^−1^.

The solar energy storage efficiency depends on the absorption ratios of NBD of all incoming light energy from the solar spectrum. As a result, a multi-NBD MOST flow device was designed to enhance energy storage efficiency (Fig. 5d). Based on the maximum and onset absorption wavelengths of NBDs (Fig. 1b–e), NBD2, NBD3, and NBD4 were chosen with a molar ratio of 1 : 1 : 1, absorbing approximately 7% of the solar energy spectrum. At a flow rate of 50 μL min^−1^, all NBDs in the multi-NBD flow MOST device can attain the PSS in both isomerization directions.

To further investigate the cyclability of the multi-NBD flow MOST device in DMF solution (cutting off the wavelength of input light below 270 nm), absorbance values were monitored using UV-Vis detectors A and B during operation (Fig. 5e and Movie S2†). These absorbance values represented the photostabilities of NBD4, NBD3, and NBD2 at three wavelengths (345 nm, 350 nm, and 380 nm). Notably, negligible fluctuations in all absorbance values were observed after 200 minutes of working cycles, thanks to the solvent-based UV shielding. This high robustness supports the two-way photoswitching flow MOST devices for durable solar energy utilization.

### MOST film

**Fig. 6:**
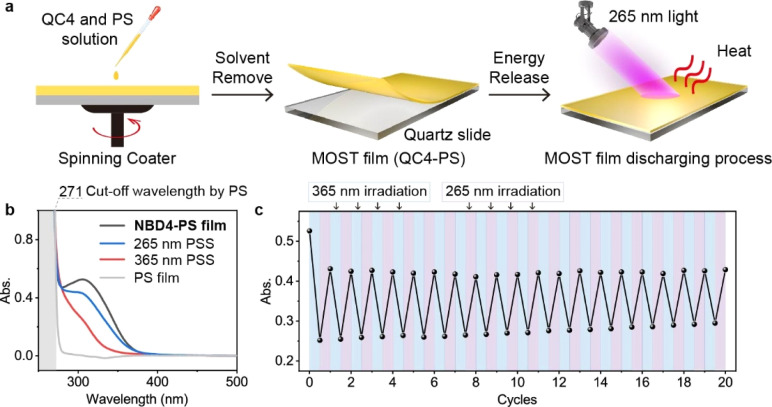
Two-way photo-switching system in a MOST film. (a) Fabrication of a MOST film. (b) UV-Vis absorption spectra of the NBD4–PS film at the 365 nm PSS and the 265 nm PSS. (c) Two-way photoswitching cycling test of the NBD4–PS film.

Efficient energy release of the NBD–QC system in a solid state remains a challenge, limited by a lack of efficient QC to NBD conversion methods. To demonstrate that two-way photoswitching NBDs can be applied not only in a MOST device in a solution but also in a MOST film in the solid state, we drop cast a QC4/polystyrene (PS) solution onto a quartz glass slide and spin coated it. This formed a MOST film with a thickness of approximately 40 μm on the quartz substrate (Fig. 6a).

The isomerization of the NBD4–PS film under 365 nm and 265 nm light irradiation was monitored by UV-Vis spectroscopy. The absorption curves at the 365 nm PSS and 265 nm PSS were similar to those in solution (Fig. 1e and 6b), confirming the achievement of the two-way photoswitching function even in the solid state. The PS film acted as a UV shield, absorbing short UV light below 262 nm and improving photostability. During alternating 365 nm and 265 nm light irradiations, the absorbance values of the 265 nm PSS at 315 nm exhibited only a slight increase, experiencing almost no degradation after 20 cycles (Fig. 6c). At the first cycle, the absorbance of NBD4 was lower than that of the original one, owing to the incomplete optical back-conversion and photodegradation. These results highlight a new practical application of the two-way photoswitching MOST system in the solid state.

## Conclusions

We have introduced a novel energy release concept based on a series of two-way photoswitching NBD–QC derivatives. The NBD molecules are designed by incorporating acceptor groups in conjunction with selected donors. The onset wavelengths of NBD derivatives are shifted into the visible light region, enabling a larger solar energy spectrum coverage (5.4%). Notably, NBD with weak acceptor and strong donor units exhibits a high back-conversion yield up to 82% under 265 nm irradiation, resulting in an efficient energy storage capacity of 312 J g^−1^. We further illustrate that with an UV shielding strategy, the photostability of NBD derivatives can be significantly improved. We successfully demonstrate the integration of two-way photoswitching NBD derivatives in both a flow device and thin film demonstrator, opening promising avenues for optically controlled energy release in NBD-based MOST systems.

## Experimental

Detailed procedures for the synthesis of NBD1, NBD2, NBD3, and NBD4 are provided in the ESI.†

### Automated MOST flow device fabrication

This automation flow MOST device consisted of two microfluidic reactors (62.5 μL and 250 μL) from Syrris, in which the NBDs in DMF solution were pumped through the device while being irradiated using 365 and 265 nm light respectively. Considering the photoisomerization rates between NBD to QC and QC to NBD, the small microfluidic reactor (62.5 μL) was set as the solar energy capture part (365 nm light) and the bigger one (250 μL) was irradiated using 265 nm light for energy release. Applying the same flow rate through the two microfluidic reactors, the irradiation time of the bigger one was four times longer than that of the small one. The real-time photoswitching yields of NBD to QC and QC to NBD were monitored using the UV-Vis detectors A and B, respectively.

### MOST film fabrication

Polystyrene (0.95 g, PS) was dissolved in tetrahydrofuran (5 mL, THF), followed by adding NBD4 (0.050 g). NBD4 was isomerized to QC4 at 365 nm irradiation in THF. A quartz glass slide (2.0 × 2.0 cm) was used as the substrate, which was washed with acetone. PS and QC4 solution (2 drops, approximately 0.1 mL) was dripped onto the quartz glass slide at a speed of 4000 revolutions per minute (rpm). The MOST film was formed on the quartz glass slide after drying at 45 °C for 60 min.

## Author contributions

L. F., C. W., and K. M.-P. conceived the idea. L. F. designed and conducted the experiments. L. F. and H. H. synthesized the molecules. L. F. and Z. W. prepared the MOST devices. K. V. M. and A. E. H.-B. performed the calculations. All the authors provided helpful discussion on this project and contributed to manuscript writing.

## Conflicts of interest

There are no conflicts to declare.

## Supplementary Material

SC-015-D4SC04247F-s001

SC-015-D4SC04247F-s002

SC-015-D4SC04247F-s003

## Data Availability

The data supporting this article have been included as part of the ESI.†
