# Supplementary material for: Two-way photoswitching norbornadiene derivatives for solar energy storage
Source: Chem Sci. 2024 Sep 26;15(43):18179–86. doi: 10.1039/d4sc04247f (PMC11474437; doi:10.1039/d4sc04247f)
Supplement: SC-015-D4SC04247F-s003 [file SC-015-D4SC04247F-s003.pdf]

Supplementary Information (SI) for Chemical Science.
This journal is © The Royal Society of Chemistry 2024

Supporting Information

Two-Way Photoswitching Norbornadiene Derivatives for Solar Energy Storage

Liang Fei,[a] Helen Hölzel,[b] Zhihang Wang,[c] Andreas Erbs Hillers-Bendtsen,[d] Adil S. Aslam,[e] Monika Shamsabadi,[e] Jialing Tan,[a] Kurt V. Mikkelsen,[d] Chaoxia Wang,*[a] and Kasper Moth-Poulsen,*[b,e,f,g]

[a] Dr. L. Fei, J. Tan, Prof. C Wang*
College of Textile Science and Engineering, Jiangnan University
1800 Lihu Road, 214122, Wuxi, China
E-mail: wangchaoxia@sohu.com

[b] Dr. H. Hölzel, Prof. K. Moth-Poulsen*
Department of Chemical Engineering, Universitat Politècnica de Catalunya, EEBE
Eduard Maristany 10-14, 08019 Barcelona, Spain
E-mail: kasper.moth-poulsen@upc.edu

[c] Dr. Z. Wang
Department of Materials Science and Metallurgy, University of Cambridge
27 Charles Babbage Rd, Cambridge CB3 0FS, U.K.

[d] Dr. A. E. Hillers-Bendtsen, Prof. K. V. Mikkelsen
Department of Chemistry, University of Copenhagen
Universitetsparken 5, 2100 Copenhagen, Denmark

[e] Dr. A. S. Aslam, M. Shamsabadi, Prof. K. Moth-Poulsen*
Department of Chemistry and Chemical Engineering, Chalmers University of Technology
Gothenburg 41296, Sweden

[f] Prof. K. Moth-Poulsen*
The Institute of Materials Science of Barcelona, ICMAB-CSIC
Bellaterra, 08193 Barcelona, Spain

[g] Prof. K. Moth-Poulsen*
Catalan Institution for Research & Advanced Studies, ICREA
Pg. Lluís Companys 23, Barcelona, Spain

Table of Contents

1. Experimental Procedures 1
1.1 Materials 1
1.2 Synthesis 1
1.3 Computational Modelling Approach 4
1.4 Characterization 4
1.5 NMR spectra 5
1.5.1 NMR of ethyl 3-bromopropiolate 5
1.5.2 NMR of ethyl (1*R*,4*S*)-3-bromobicyclo[2.2.1]hepta-2,5-diene-2-carboxylate 6
1.5.3 NMR of NBD1 7
1.5.4 NMR of NBD2 8
1.5.5 NMR of 3-(4-methoxyphenyl)-2-propynenitrile 9
1.5.6 NMR of NBD3 9
1.5.7 NMR of NBD4 10
2. Results and Discussion 12
2.1 Two-Way Photoswitching Properties 12
2.1.1 Theory calculation 12
2.1.2 Solar energy absorption utilization 13
2.1.3 Photoisomerization kinetics 13
2.1.4 Photoconversion yields 14
2.1.5 Photoisomerization quantum yields (Φ) calculation 18
2.1.6 Thermal back conversion 23
2.2 Photostability and Photodegradation 27
2.2.1 Photodegradation of NBD derivatives in ^{1}H NMR spectra 27
2.2.2 Photodegradation of NBD derivatives in HRMS 28
2.2.3 Photodegradation comparison of optical and thermal back-conversion 30
2.2.4 UV-Vis absorption spectra after photodegradation 31
2.3 Solar Energy Storage and Release by Light 32
2.4 Automation Flow MOST Device 33
3. Cartesian Coordinates 34
References 47

1. Experimental Procedures

1.1 Materials

All reagents were purchased from commercial suppliers and used as received unless noted otherwise.

1.2 Synthesis

Ethyl 3-bromopropiolate molecule was synthesized as following:

To a solution of alkyne (1 eq.) in acetone (0.2 mmol mL^{-1}) was added NBS (1.1 eq.) and $AgNO_3$ (10 mol%) at room temperature with magnetic stirring. After 2 h, the reaction mixture was diluted with hexanes (100 mL) and filtered off the crystals formed. The filtrate was concentrated under reduced pressure and passed through a pad of silica gel using hexanes as an eluent. The filtrate was collected and evaporated under reduced pressure to afford a pure colorless oil of ethyl 3-bromopropiolate with yield of 86%. ^{1}H NMR (400 MHz, $CDCl_3$) δ 4.22 (q, J = 7.1 Hz, 2H), 1.29 (t, J = 7.1 Hz, 3H). ^{13}C NMR (100 MHz, $CDCl_3$) δ 152.47, 77.33, 77.01, 76.70, 72.77, 62.47, 52.40, 13.94, 13.92. HRMS (ESI) m/z: $[M + H]^+$ calcd for $C_5H_5BrO_2$, 176.9545; found, 176.9529.

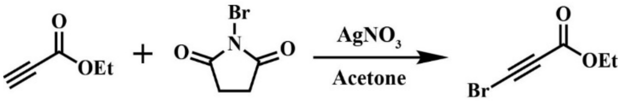

Scheme S1. Synthesis of ethyl 3-bromopropiolate.

Ethyl (1*R*,4*S*)-3-bromobicyclo[2.2.1]hepta-2,5-diene-2-carboxylate was synthesized as following:

Cyclopentadiene was distilled by cracking dicyclopentadiene and storing at −80 °C prior to use. A vial suitable for microwave reactions was charged with cyclopentadiene (3 eq.), ethyl 3-bromopropiolate (1 eq.), and BHT (Butylated hydroxytoluene) without the solvent. The vial was sealed at 60 °C for 24 h. The resulting reaction mixture was purified on a Biotage Isolera One system using a Biotage KP-sil 10 g column (ethyl acetate/petroleum spirit 1:9) to afford a light yellow oil of ethyl (1*R*,4*S*)-3-bromobicyclo[2.2.1]hepta-2,5-diene-2-carboxylate with yield of 79%. ^{1}H NMR (400 MHz, $CDCl_3$) δ 6.85 (dd, J = 5.1, 3.0 Hz, 1H), 6.79 (dd, J = 5.2, 2.9 Hz, 1H), 4.21–4.07 (m, 2H), 3.94 (s, 1H), 3.62 (s, 1H), 2.25 (d, J = 6.7 Hz, 1H), 2.06 (d, J = 6.7 Hz, 1H), 1.25 (t, J = 7.1 Hz, 3H). ^{13}C NMR (100 MHz, $CDCl_3$) δ 163.68, 148.39, 143.00, 142.05, 140.38, 71.70, 61.62, 60.42, 52.02, 14.23. HRMS (ESI) m/z: $[M + H]^+$ calcd for $C_{10}H_{11}BrO_2$, 243.0015; found, 243.0017.

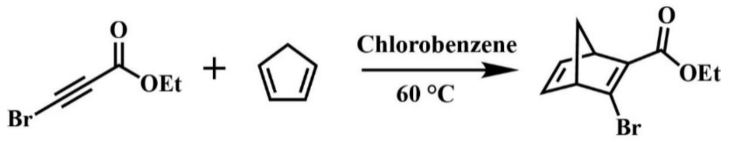

Scheme S2. Synthesis of ethyl (1*R*,4*S*)-3-bromobicyclo[2.2.1]hepta-2,5-diene-2-carboxylate.

NBD1 was synthesized as following:

To a dry degassed solution of ethyl (1*R*,4*S*)-3-bromobicyclo[2.2.1]hepta-2,5-diene-2-carboxylate (1 eq.) in THF (10 mL) was added (4-methoxyphenl)bronic acid (1.1 eq.), cesium fluoride (3 eq), tris(dibenzylideneacetone)dipalladium(0) (0.050 eq.) and a solution of tritert-butylphosphine (1.5 eq., 1 mol mL^{-1} in toluene, 1.5 mmol). The mixture was stirred under a nitrogen atmosphere for 2.5 h at ambient temperature and then heated to 60 °C for 40 h. The reaction mixture was allowed to cool to room temperature and was filtered through a pad of Celite®, washing with dichloromethane (50 mL). The solvent of the filtrate was removed under reduced pressure and

purified on a Biotage Isolera One system using a Biotage KP-sil 100 g column (ethyl acetate /hexane 1:5) to afford a yellow oil of NBD1 with yield of 18%.^{1}H NMR (400 MHz, CD_3CN) δ 7.61 – 7.53 (m, 2H), 6.96 (t, J = 2.0 Hz, 2H), 6.95 – 6.91 (m, 2H), 4.17 – 4.05 (m, 2H), 4.03 – 3.96 (m, 1H), 3.89 (tt, J = 2.4, 1.3 Hz, 1H), 3.82 (s, 3H), 2.18 (t, J = 1.7 Hz, 1H), 2.00 (dt, J = 6.7, 1.6 Hz, 1H), 1.21 (t, J = 7.1 Hz, 3H). ^{13}C NMR (100 MHz, CD_3CN) δ 172.05, 166.93, 160.08, 143.38, 140.87, 137.10, 129.69, 117.34, 113.06, 69.80, 59.78, 58.08, 54.97, 53.02, 13.52. HRMS (ESI) m/z: $[M + H]^+$ calcd for $C_{17}H_{18}O_3$, 271.1329; found, 271.1311. Melting point (M.P.) 20 °C

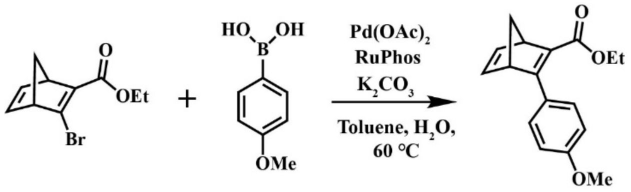

Scheme S3. Synthesis of NBD1.

NBD2 was synthesized as following:

A vial suitable for microwave reactions was charged with cyclopentadiene (1.98 g, 30 mmol, 3 eq.), trifluoroacetyl 3-bromopropiolate (2.28 g, 10 mmol, 1 eq.), and BHT without the solvent. The vial was sealed at 100 °C for 24 h. The resulting reaction mixture was purified on a Biotage Isolera One system using a Biotage KP-sil 10 g column (ethyl acetate/petroleum spirit 1:7) to afford a yellow oil of NBD2 with yield of 57%.^{1}H NMR (400 MHz, CD_3CN) δ 7.79–7.71 (m, 2H), 6.97–6.85 (m, 4H), 4.18 (s, 1H), 3.96 (s, 1H), 3.85 (s, 3H), 2.23 (dt, J = 7.1, 1.8 Hz, 1H), 2.16 (dt, J = 7.0, 1.6 Hz, 1H). ^{13}C NMR (100 MHz, CD_3CN) δ 161.84, 143.62, 140.13, 130.63, 127.04, 117.33, 113.36, 68.97, 58.85, 55.18, 51.90, 51.87. HRMS (ESI) m/z: $[M + H]^+$ calcd for $C_{16}H_{13}F_3O_2$, 295.0940; found, 295.0951. M.P. 5 °C

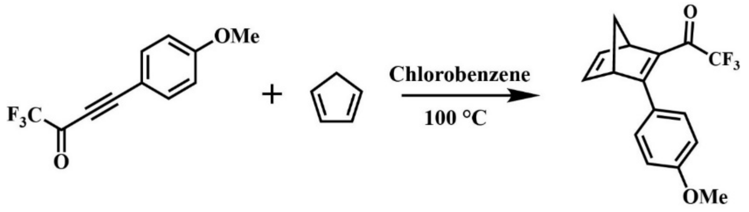

Scheme S4. Synthesis of NBD2.

NBD3 was synthesized as following:

$POCl_3$(22 mL) in DMF was added into a three-neck round bottom flask charged with fresh under N_2 and cooled with an ice bath. A solution of 4- methoxyacetophenone (30 g ,0.20 mol) was prepared in DMF (40 mL) and was added under N_2 dropwise to the reaction, followed by a further addition of DMF (20 ml). The reaction was removed from the ice bath and placed into an oil bath, heating to 60 °C for 3 h. The reaction mixture was poured into a saturated NaOAc solution (1 L) and was stirred for 1 hour at room temperature, which gave an orange precipitate. The precipitate was filtered off and washed with deionised water.

The precipitate was dissolved in the DCM, followed by adding elemental iodine (35 g). The reaction was placed in an ice bath and 25% aqueous NH_3 (295 mL) was added portion wise over 15 min. The ice bath was removed , and the solution was stirred at room temperature for 5 h. The saturated $Na_2S_2O_3$ solution (500ml) was added to obtain a yellow solution. The phases were mixed in the separation funnel and separated, with the aqueous phase being extracted with DCM (400 ml). The organic layers were dried over $MgSO_4$ and filtered , and the solvent was removed.

The residue was dissolved in THF (480 mL), and a NaOH solution (22g in 100mL) was added portion wise over 5 min. After stirring for 60 h, a saturated $NaHCO_3$ solution (500 mL) was added and the THF phase was concentrated in vacuo. The residue was taken up in DCM and washed with aqueous phase, and the organic layers were dried over Na_2SO_4. After filtration , the solvent was removed in

vacuo , and and the residue was purified on a Biotage Isolera One system using a Biotage KP-sil 100 g column (petroleum ether:DCM=1:1) to obtain 3-(4-methoxyphenyl)-2-propynenitrile with yield of 41%. ^{1}H NMR (400 MHz, $CDCl_3$) δ 7.54 (d, *J* = 8.3 Hz, 2H), 6.89 (d, *J* = 8.6 Hz, 2H), 3.84 (s, 3H). HRMS (ESI) m/z: $[M + H]^+$ calcd for $C_7H_{10}NO$, 158.0600; found, 158.0588.

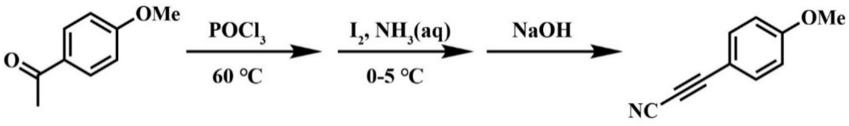

Scheme S5. Synthesis of 3-(4-methoxyphenyl)-2-propynenitrile.

A vial suitable for microwave reactions was charged with cyclopentadiene (1.98 g, 30 mmol, 3 eq.), 3-(4-methoxyphenyl)-2-propynenitrile (1.57 g, 10 mmol, 1 eq.), and BHT without the solvent. The vial was sealed at 100 °C for 24 h. The resulting reaction mixture was purified on a Biotage Isolera One system using a Biotage KP-sil 10 g column (ethyl acetate/petroleum spirit 1:6) to afford a light yellow solid of NBD3 with yield of 51%. ^{1}H NMR (400 MHz, CD_3CN) δ 7.78 – 7.68 (m, 2H), 7.07 – 6.99 (m, 2H), 6.96 (dd, *J* = 2.9, 0.8 Hz, 1H), 6.93 – 6.88 (m, 1H), 4.17 (ddtd, *J* = 3.2, 2.4, 1.6, 0.7 Hz, 1H), 3.91 (dq, *J* = 3.2, 1.0 Hz, 1H), 3.84 (s, 3H), 2.23 (dt, *J* = 6.9, 1.6 Hz, 1H), 2.12 (dt, *J* = 6.9, 1.6 Hz, 1H). ^{13}C NMR (100 MHz, CD_3CN) δ 170.71, 161.19, 143.10, 140.41, 128.13, 125.87, 118.68, 117.36, 114.31, 113.78, 70.56, 55.17, 54.72, 54.15. HRMS (ESI) m/z: $[M + H]^+$ calcd for $C_{15}H_{13}NO$, 224.1070; found, 224.1056. M.P. 39 °C

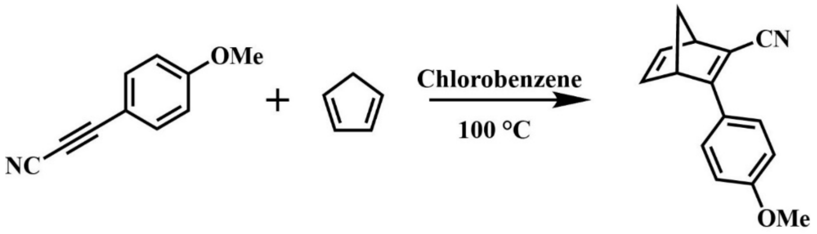

Scheme S6. Synthesis of NBD3.

NBD4 was synthesized as following:

To a dry degassed solution of ethyl (1*R*,4*S*)-3-bromobicyclo[2.2.1]hepta-2,5-diene-2-carboxylate (1 eq.) in THF (10 mL) was added (4-acetaminophenyl)boronic acid (1.1 eq.), cesium fluoride (3 eq), tris(dibenzylideneacetone)dipalladium(0) (0.050 eq.) and a solution of tritert-butylphosphine (1.5 eq., 1M in toluene, 1.5 mmol). The mixture was stirred under a nitrogen atmosphere for 2.5 h at ambient temperature and then heated to 60 °C for 40 h. The reaction mixture was allowed to cool to room temperature and was filtered through a pad of Celite®, washing with dichloromethane (50 mL). The mixture was concentrated under reduced pressure and purified on a Biotage Isolera One system using a Biotage KP-sil 100 g column (ethyl acetate /hexane 1:4) to afford a light yellow solid of NBD4 with yield of 18%. ^{1}H NMR (400 MHz, $CDCl_3$) δ 7.54 (d, *J* = 8.1 Hz, 2H), 7.49 (d, *J* = 8.9 Hz, 2H), 7.18 (s, 1H), 6.99–6.93 (m, 1H), 6.92–6.85 (m, 1H), 4.13 (q, *J* = 6.8 Hz, 2H), 4.04 (s, 1H), 3.83 (s, 1H), 2.20 (s, 1H), 2.18 (s, 3H), 2.03 (d, *J* = 6.7 Hz, 1H), 1.23 (t, *J* = 7.1 Hz, 3H). ^{13}C NMR (101 MHz, $cdcl_3$) δ 168.32, 165.66, 143.67, 140.61, 138.20, 131.35, 128.85, 118.66, 77.33, 77.01, 76.70, 70.28, 60.12, 58.29, 53.00, 24.68, 14.21. HRMS (ESI) m/z: $[M + H]^+$ calcd for $C_{18}H_{19}NO_3$, 298.1438; found, 298.1435. M.P. 45 °C

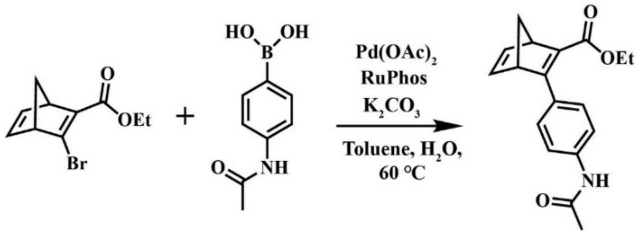

Scheme S7. Synthesis of NBD4.

1.3 Computational Modelling Approach

Initially, the lowest energy conformer and an approximate transition state of each NBD/QC system was generated using the extended tight binding (xTB) screening procedure developed in previous studies[1, 2]. In short, a systematic conformational search is performed using RDKit[3] and subsequently the structures of NBD and QC are optimized using GFN2-xTB[4] and the lowest energy conformer is selected. The transition state guess in the approximated as the highest energy geometry from a 20 point GFN2-xTB scan that symmetrically open the bonds of the QC molecule to form NBD.

Using these geometries, we performed geometry optimization and subsequent frequency calculations of each compound using density functional theory (DFT) at the M06-2X/def2-SVPD level of theory[5, 6]. To scrutinize the excited states of each system, we initially performed time-dependent DFT calculations to determine the 50 lowest vertical excitation energies and associated oscillator strengths using CAM-B3LYP[7]/def2-SVPD. This data was then converted to UV-Vis absorption spectra by convoluting each excitation energy to Lorentzian functions using a width of 0.4 eV. To further probe the topology of the first excited state, we optimized the minimum energy conical intersection (MECI) between the S_1 and S_0 states using CAM-B3LYP/def2-SVPD, and we performed single point calculations on interpolations between the NBD and MECI as well as the QC and MECI to get a qualitative mapping of the energy path through which NBD is converted to QC and vice versa. The interpolations were obtained using the independent pair potential model[8].

All DFT calculations were run in Orca 5[9] with solvation in acetonitrile included via the conductor like polarizable continuum model[10] and the large DEF3GRID for integration of the exchange-correlation functional.

1.4 Characterization

^{1}H NMR (400 MHz) and ^{13}C NMR (100 MHz) spectra were recorded on a Varian 400 MHz instrument using the residual solvent as the internal standard ($CDCl_3$, ^{1}H 7.26 ppm and ^{13}C 77.16 ppm or CD_3CN, ^{1}H 1.94 ppm, ^{13}C 1.32 ppm, 118.26 ppm) at 25 °C. The high resolution mass spectra (HRMS) were obtained by an Agilent 1260 Infinity fitted with an Agilent 6120 quadrupole using APCI mode for ionisation. All UV-Vis absorbance analyses were performed using a Cary 100 UV-Vis spectrophotometer or a PerkinElmer UV-Vis-NIR Lambda 950 spectrophotometer. All the tests were performed at 25 °C, unless the specific temperatures were mentioned. The temperature control during thermal back-conversion was achieved with Peltier temperature control. All photoswitching experiments were performed using a Thorlabs LED with wavelengths of 250-455 nm with the light intensity of 20 mW cm^{-2}. Mettler Toledo DSC 2 apparatus was used to collect the Differential scanning calorimetry (DSC) curves at a heating/cooling rate of 5 °C min^{-1} under N_2 atmosphere. The melting points of NBDs were also determined by the DSC, and the onset temperature of the melting peak is reported as the melting point.

1.5 NMR spectra

1.5.1 NMR of ethyl 3-bromopropiolate

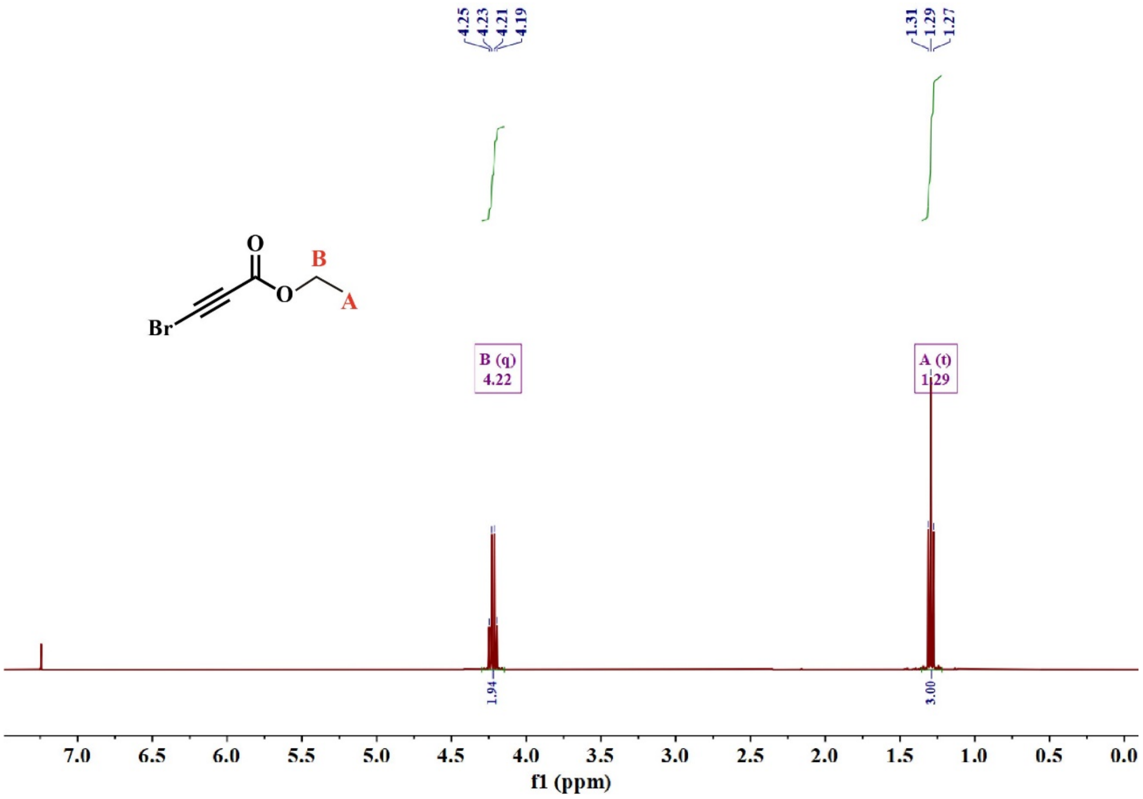

^{1}H NMR of ethyl 3-bromopropiolate.

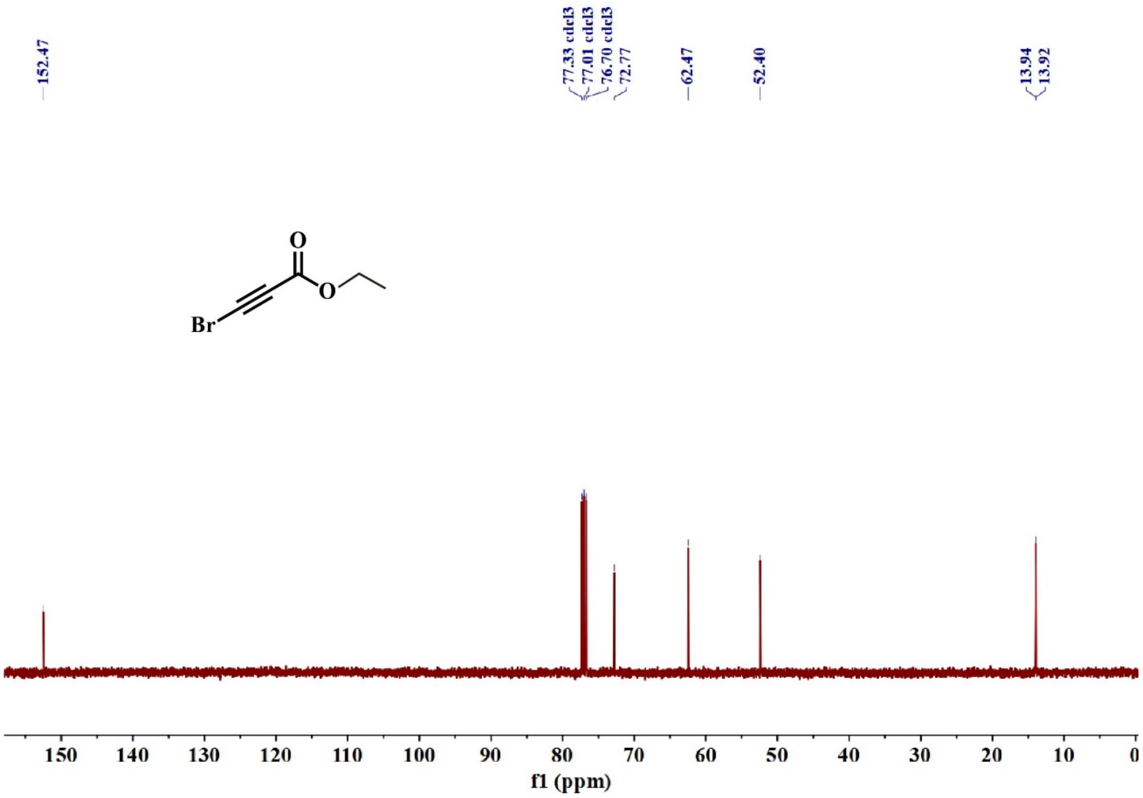

^{13}C NMR of ethyl 3-bromopropiolate.

1.5.2 NMR of ethyl (1*R*,4*S*)-3-bromobicyclo[2.2.1]hepta-2,5-diene-2-carboxylate

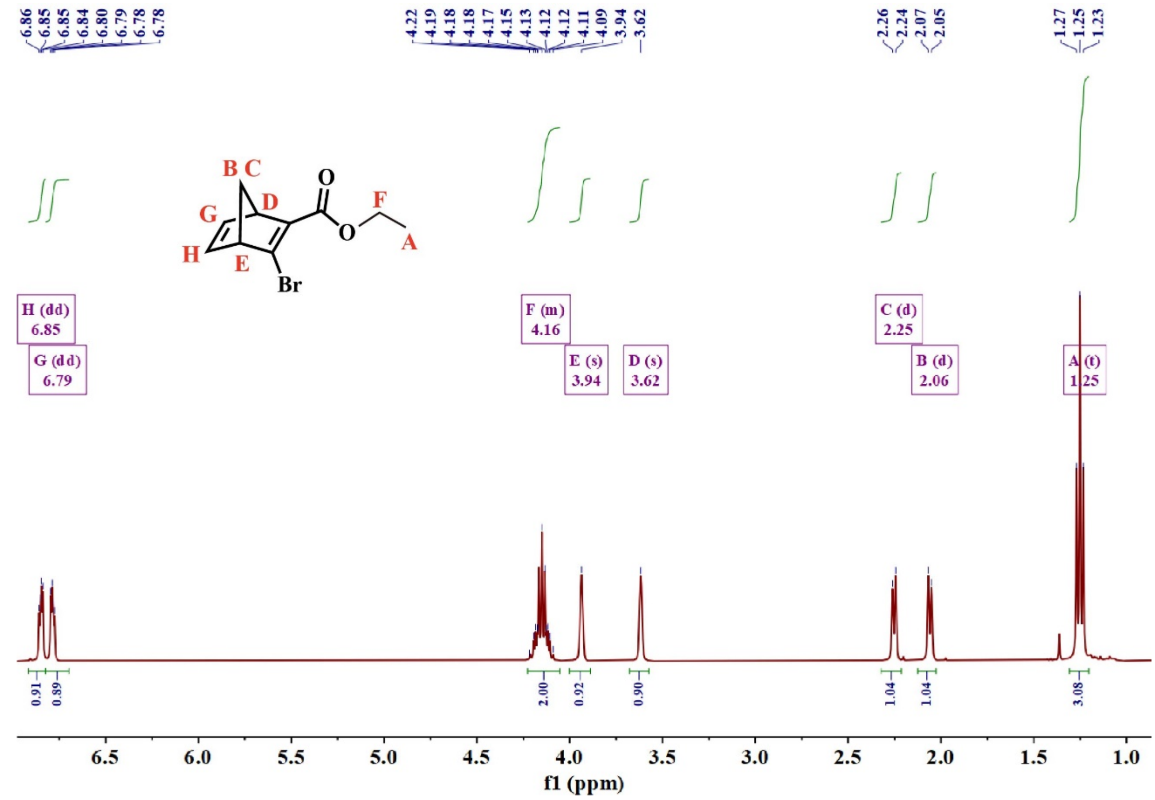

^{1}H NMR of ethyl (1*R*,4*S*)-3-bromobicyclo[2.2.1]hepta-2,5-diene-2-carboxylate.

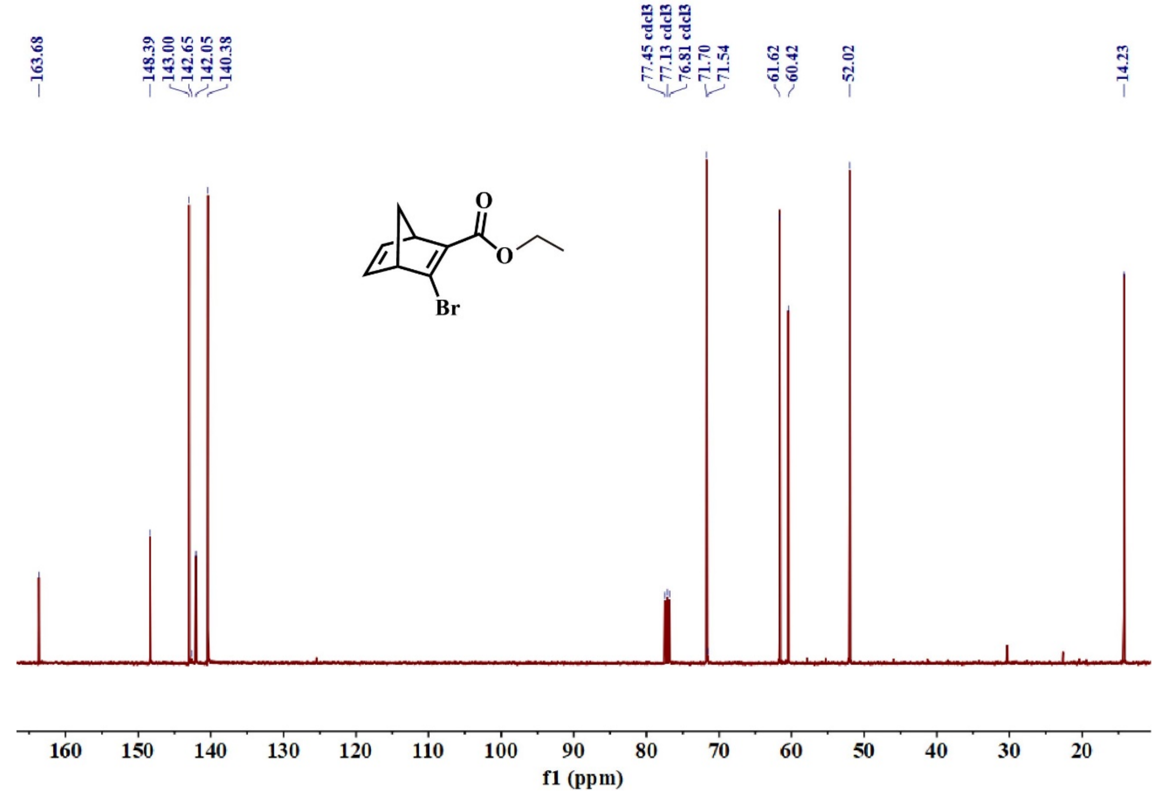

^{13}C NMR of ethyl (1*R*,4*S*)-3-bromobicyclo[2.2.1]hepta-2,5-diene-2-carboxylate.

1.5.3 NMR of NBD1

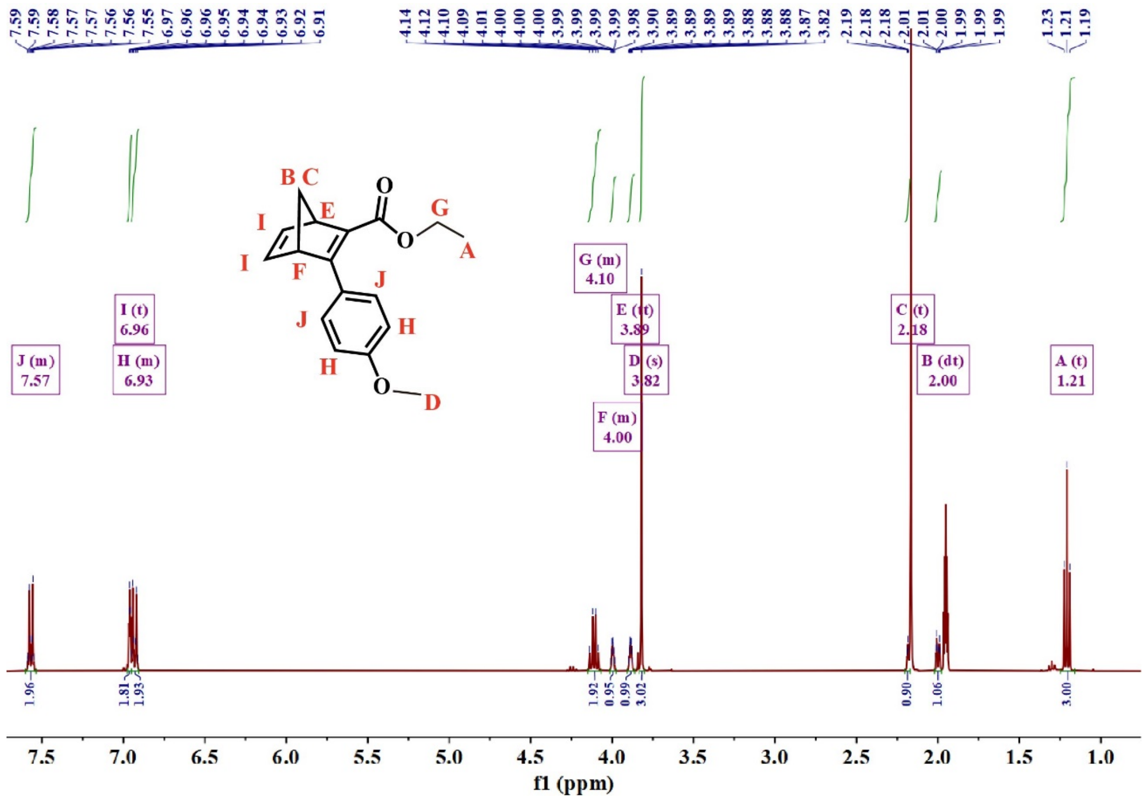

¹H NMR of NBD1.

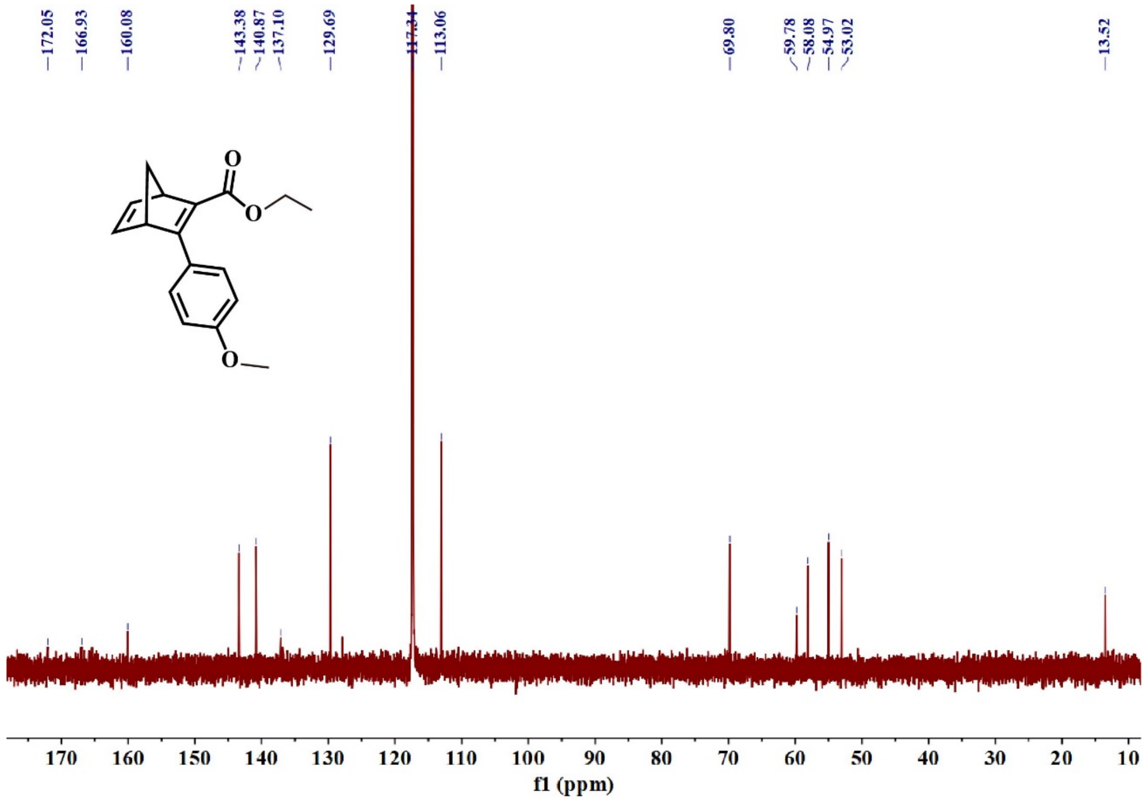

¹³C NMR of NBD1.

1.5.4 NMR of NBD2

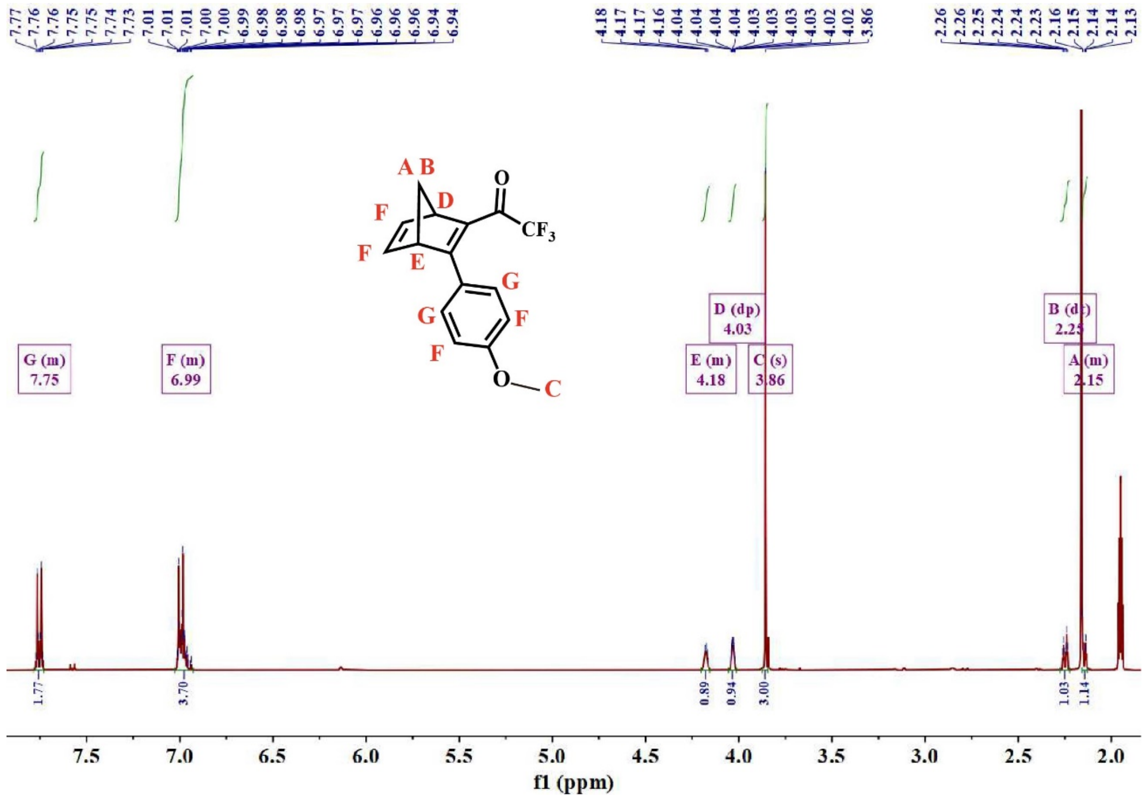

^{1}H NMR of NBD2.

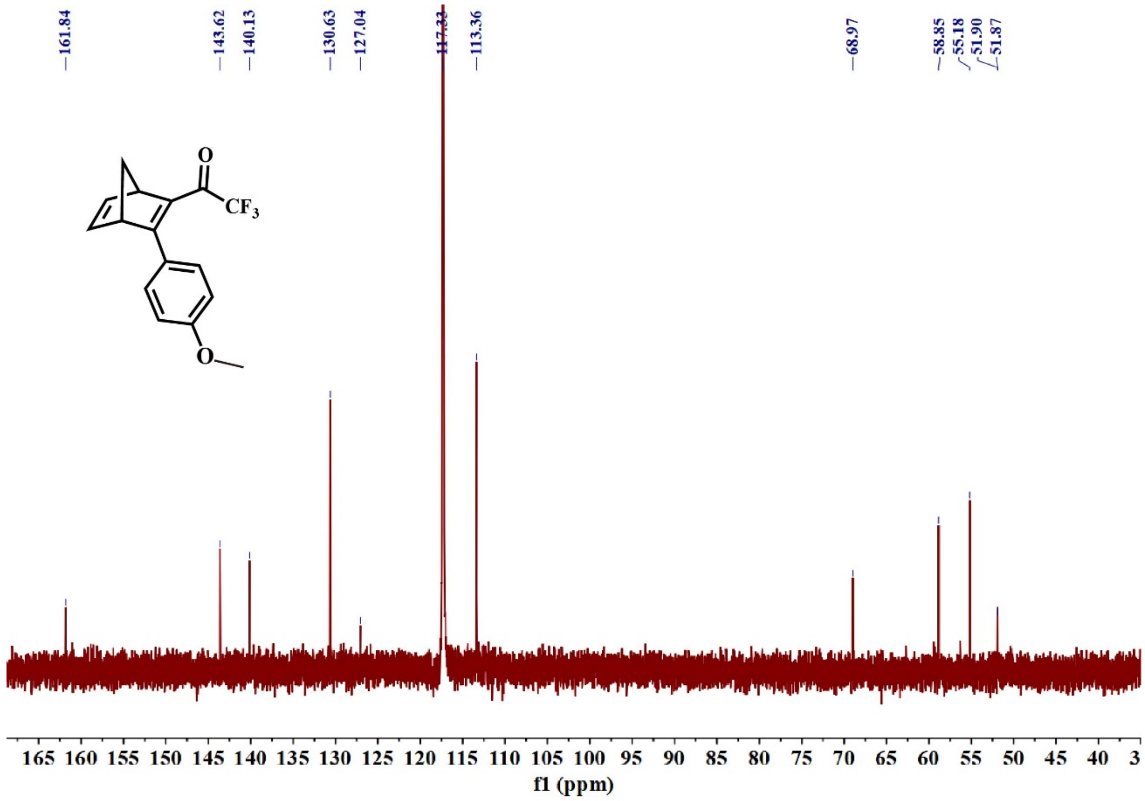

^{13}C NMR of NBD2.

1.5.5 NMR of 3-(4-methoxyphenyl)-2-propynenitrile

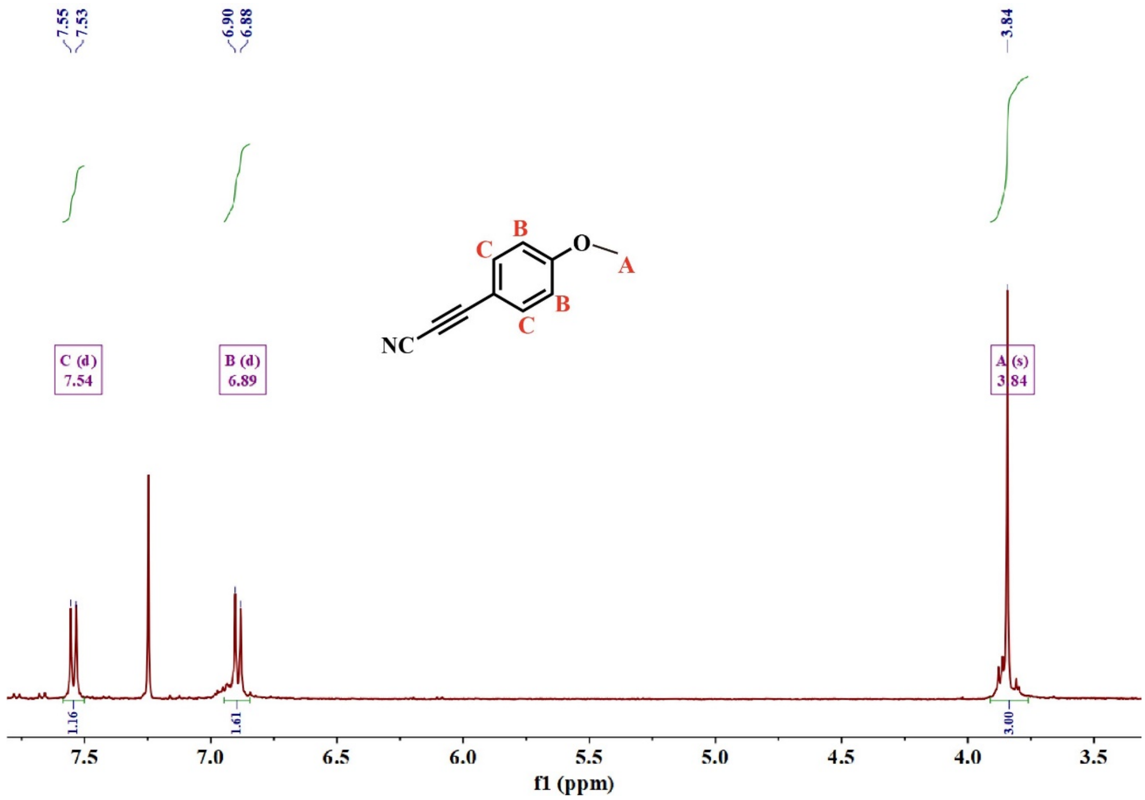

^1H NMR of 3-(4-methoxyphenyl)-2-propynenitrile.

1.5.6 NMR of NBD3

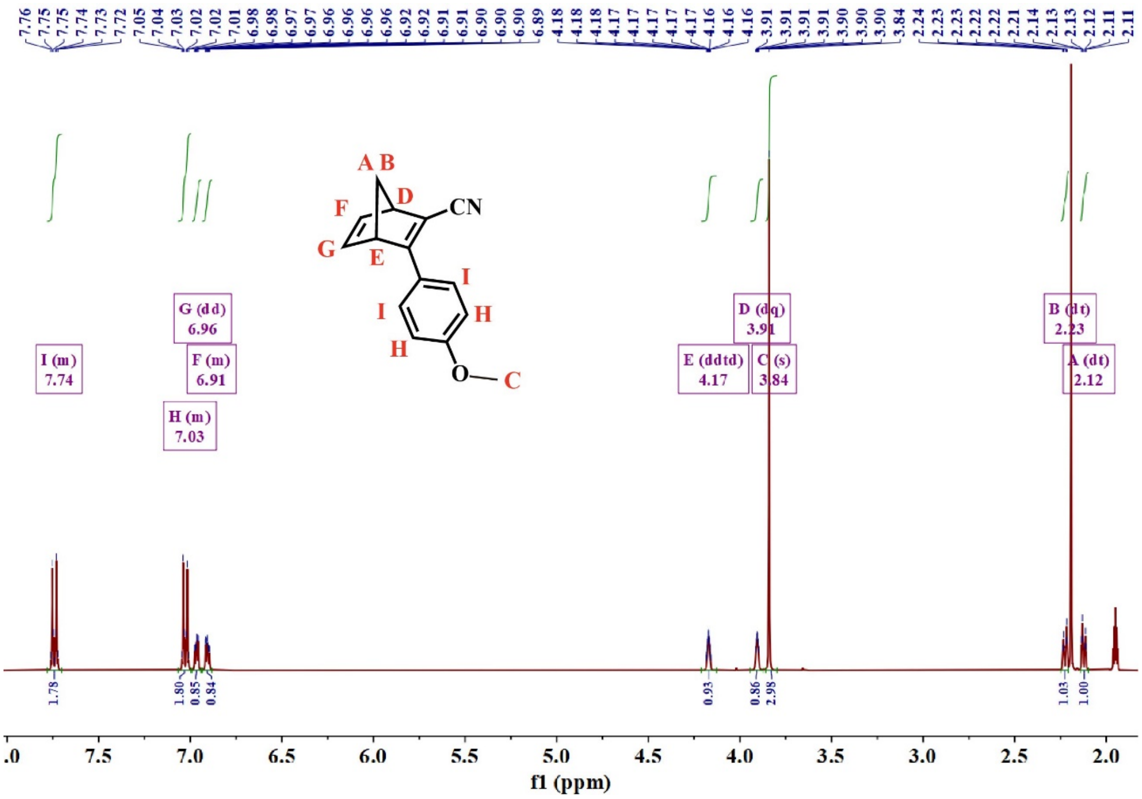

^{1}H NMR of NBD3.

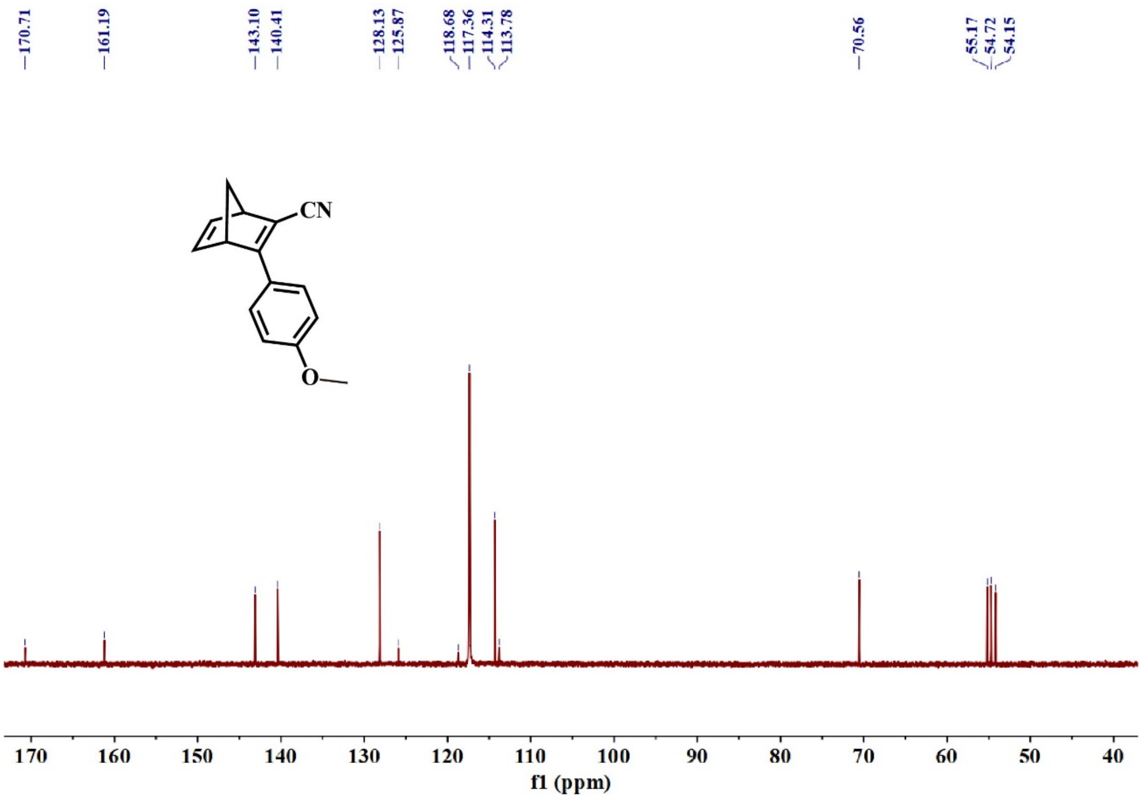

^{13}C NMR of NBD3.

1.5.7 NMR of NBD4

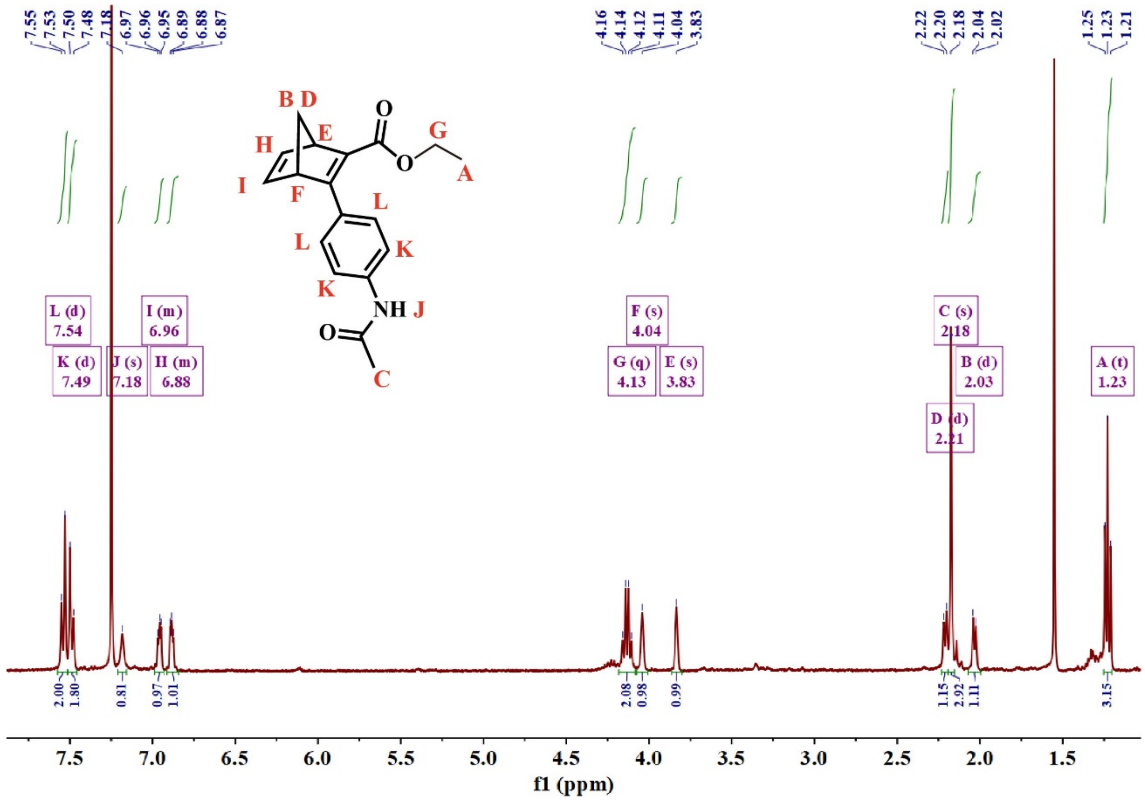

^{1}H NMR of NBD4.

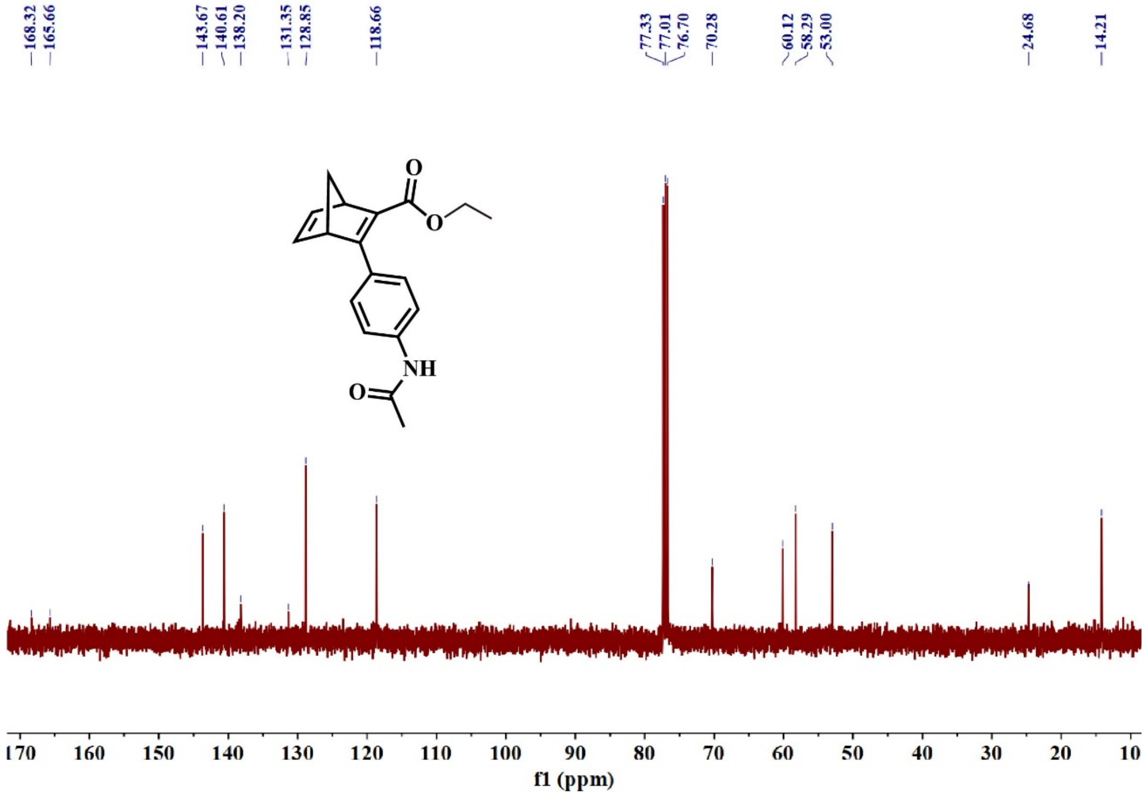

^{13}C NMR of NBD4.

2. Results and Discussion

2.1 Two-Way Photoswitching Properties

2.1.1 Theory calculation

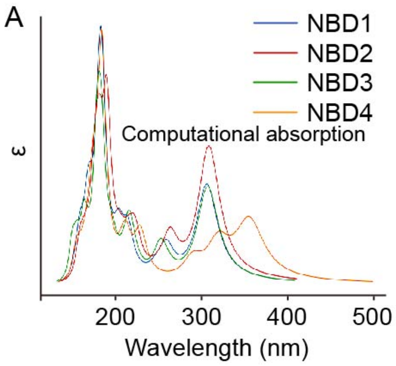

Fig. S1. UV-Vis absorption spectra of NBDs and QCs via Computational Modelling Approach.

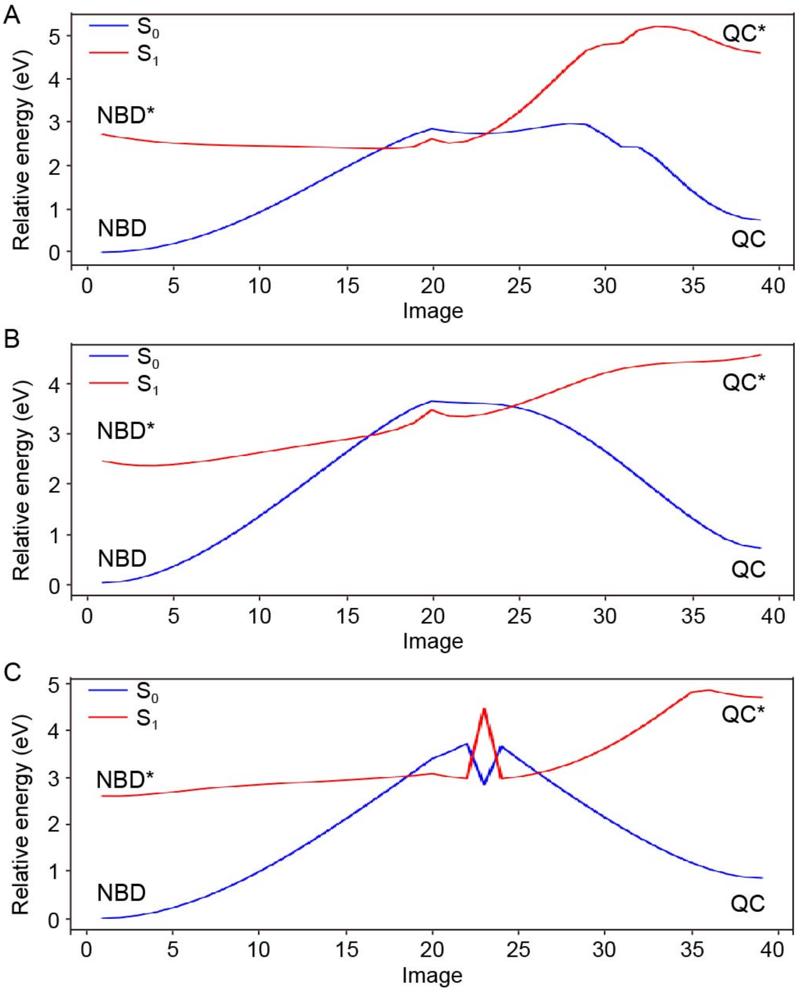

Fig. S2. Photoisomerization paths between (A) NBD1 and QC1, (B) NBD2 and QC2, (C) NBD3 and QC3, via the minimum energy conical intersection.

2.1.2 Solar energy absorption utilization

To calculate the maximum solar energy absorption utilization for NBD derivatives, the following equation can be addressed[11, 12]:

$$\eta = \frac{\int_0^{\lambda_{onset}} E_{AM1.5}(\lambda) \cdot d\lambda}{\int E_{AM1.5}(\lambda) \cdot d\lambda} \qquad (S1)$$

Where $E_{AM1.5}(\lambda)$ presents the solar spectrum energy; λ_{onset} corresponds to onset wavelength of absorption peak in UV-Vis absorption spectra (Fig. 1).

The spectrum of the standard sun irradiation (AM 1.5) was presented in Fig. S1. The black curve represented the solar spectrum AM 1.5 in a wavelength of 280-500 nm. The purple, red, blue, and green parts showed the solar energy absorption by NBD1, NBD2, NBD3, and NBD4, respectively. The higher λ_{onset} indicated the light absorption of NBD derivatives overlapped larger sun spectrum, leading to a higher solar energy utilization efficiency.

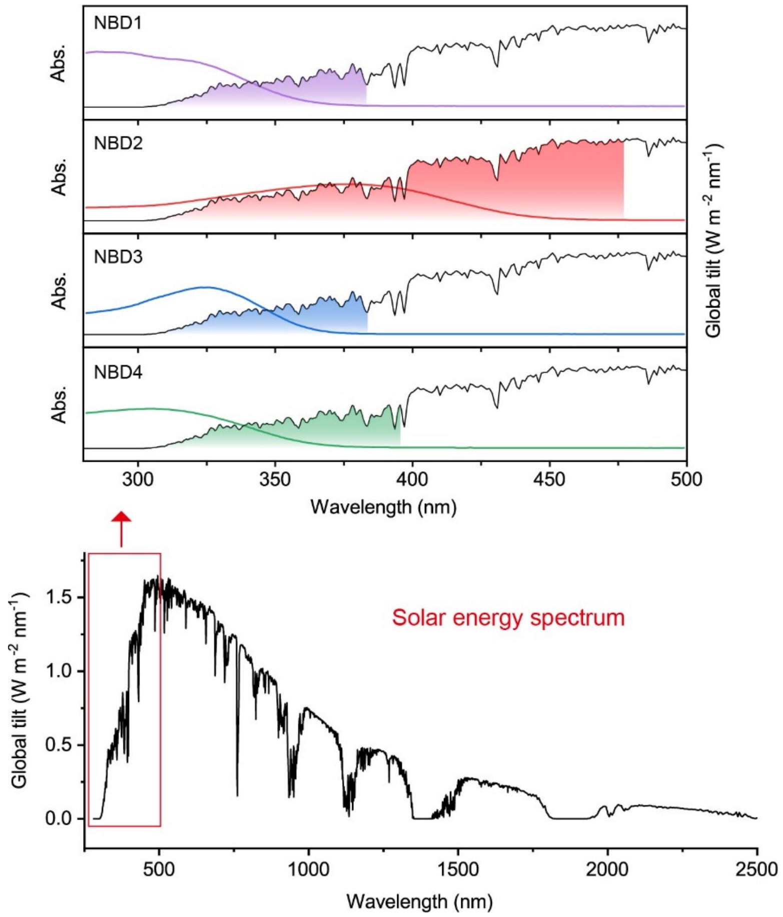

Fig. S3. Solar energy absorption utilization of NBD1, NBD2, NBD3, and NBD4.

2.1.3 Photoisomerization kinetics

The photoisomerization kinetics in both photoswitching direction of NBD-to-QC and QC-to-NBD fitted a first-order reaction model under 365 nm and 265 nm irradiation, respectively. All the curves are fitted by first-order kinetics equation as following:

$$y = -Ae^{-k} + C \qquad (S2)$$

Where *k* presents the first-order kinetics rate constant.

Irradiation at 365 nm presented the characteristic decrease of the NBD4 absorption band in the UV and Vis regions, which was associated with conversion to QC (Fig. S2A and B). The absorption spectra of the quadricyclane isomers were back converted under a 265 nm irradiation (Fig. S2C and D), showing a two-way photoswitching function for designed NBD derivatives.

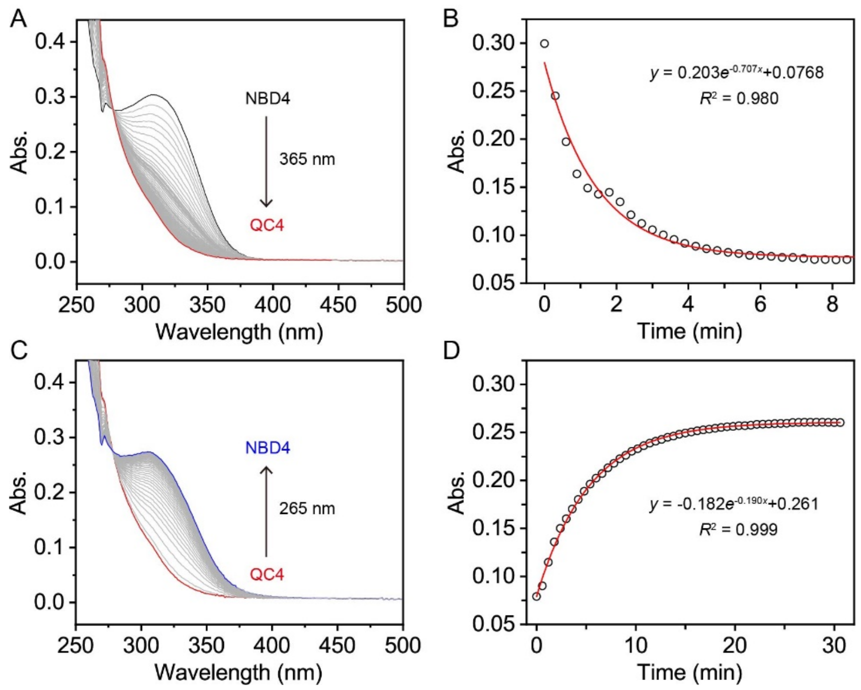

Fig. S4. UV–Vis absorption spectra in MeCN solution (0.05 mM L^{-1}) of NBD4 under (A) 365 nm and (C) 265 nm light irradiation. The photoisomerization conversion of (B) NBD-to-QC and (D) QC-to-NBD, followed as the absorbance at 315 nm.

2.1.4 Photoconversion yields

The photoconversion yields of NBD derivatives under 365 nm irradiation were calculated in ^{1}H NMR spectra via integration of peaks representing NBD and QC isomers. After photoisomerization of NBD-to-QC, the protons presented clear shifts. For example, the proton peaks of NBD3 on benzene were at 7.75 and 7.05 ppm at NBD state, while those peaks converted to 7.24 and 6.86 ppm at QC state (Fig. S3).

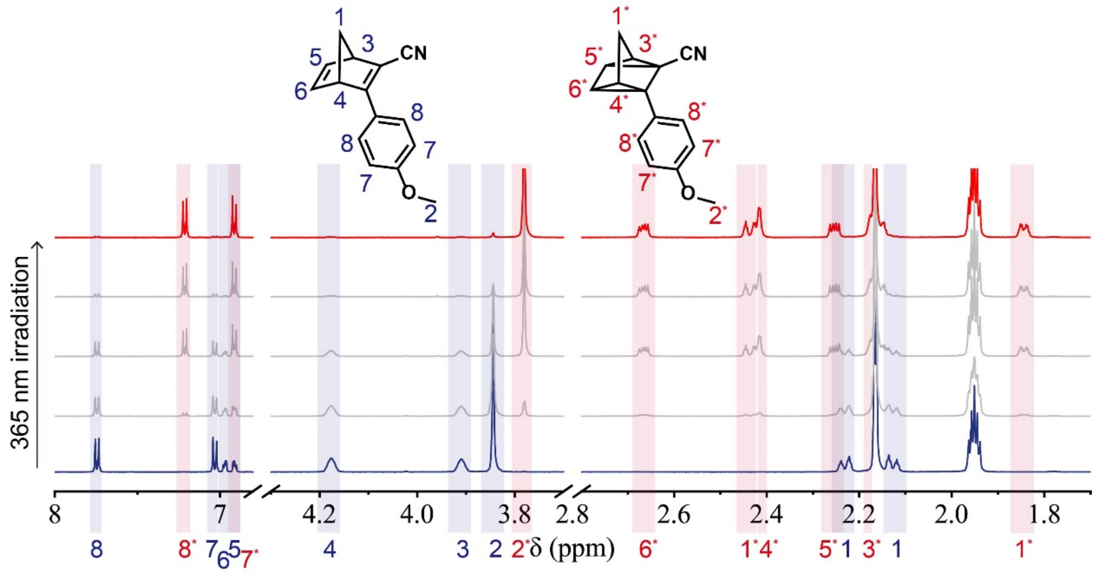

Fig. S5. ^{1}H NMR spectra of the progressive photoconversion of NBD3-to-QC3 under 365 nm light irradiation (solvent: CD_3CN).

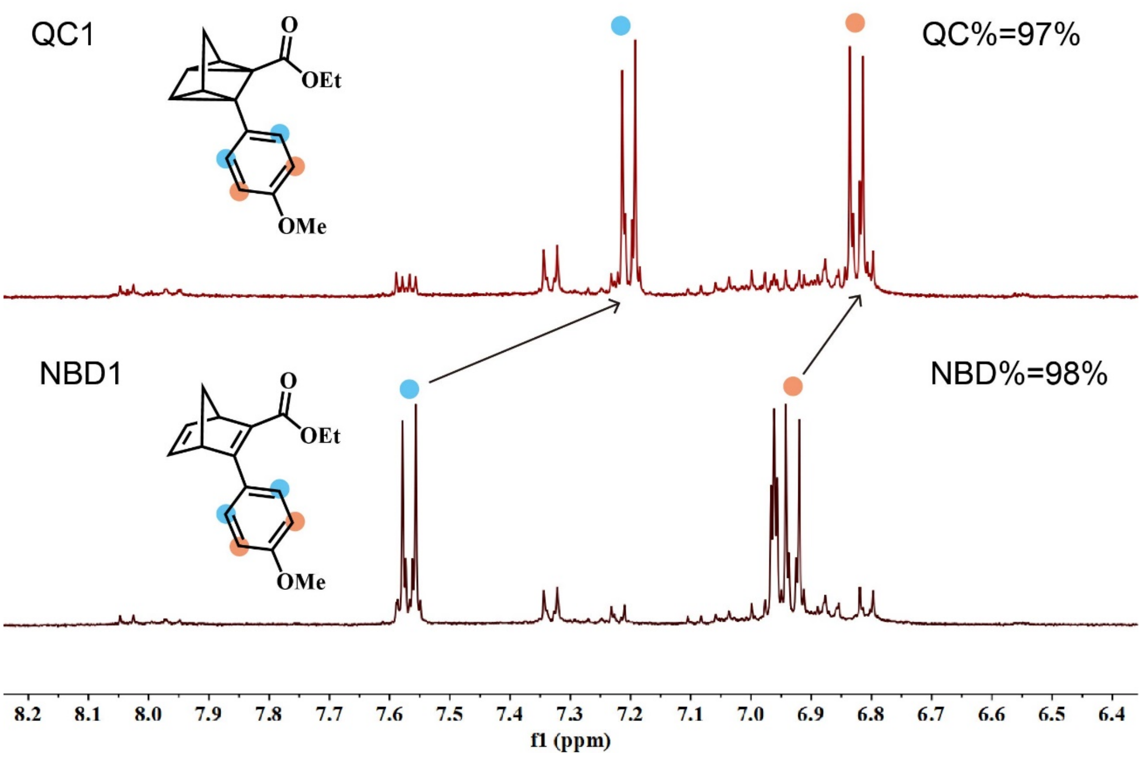

Fig. S6. ^{1}H NMR spectra and photoconversion yield of NBD1 under 365 nm light irradiation (solvent: CD_3CN).

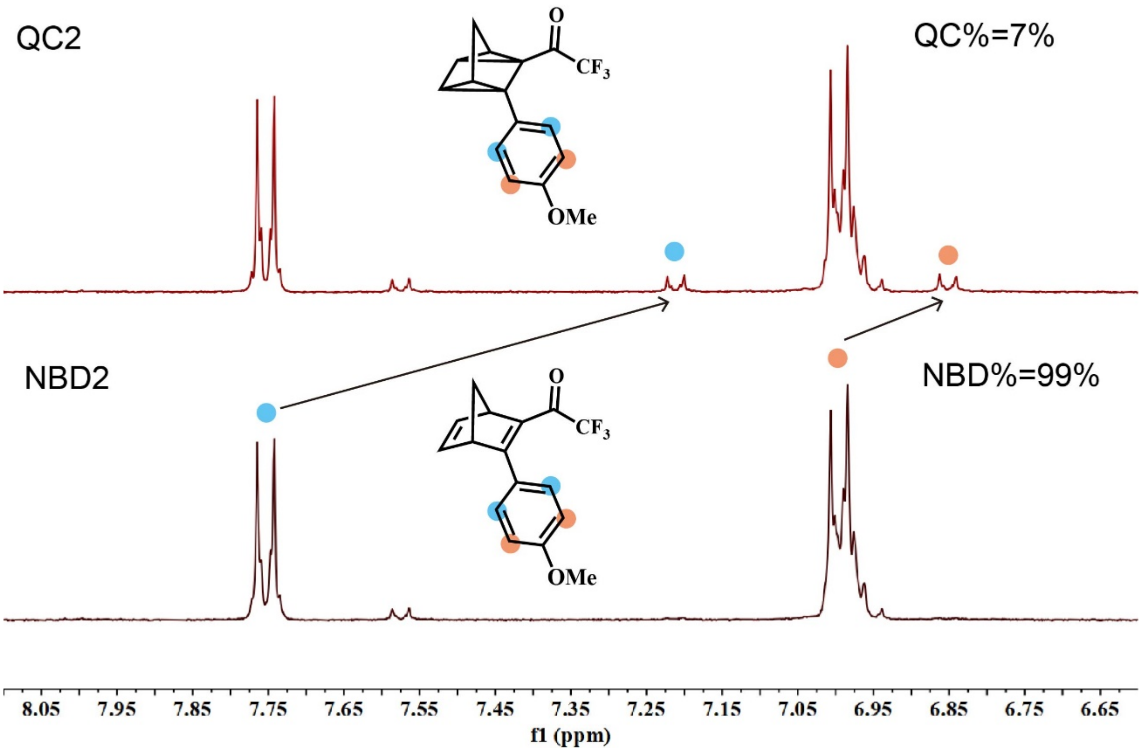

Fig. S7. ^{1}H NMR spectra and photoconversion yield of NBD2 under 365 nm light irradiation (solvent: CD_3CN).

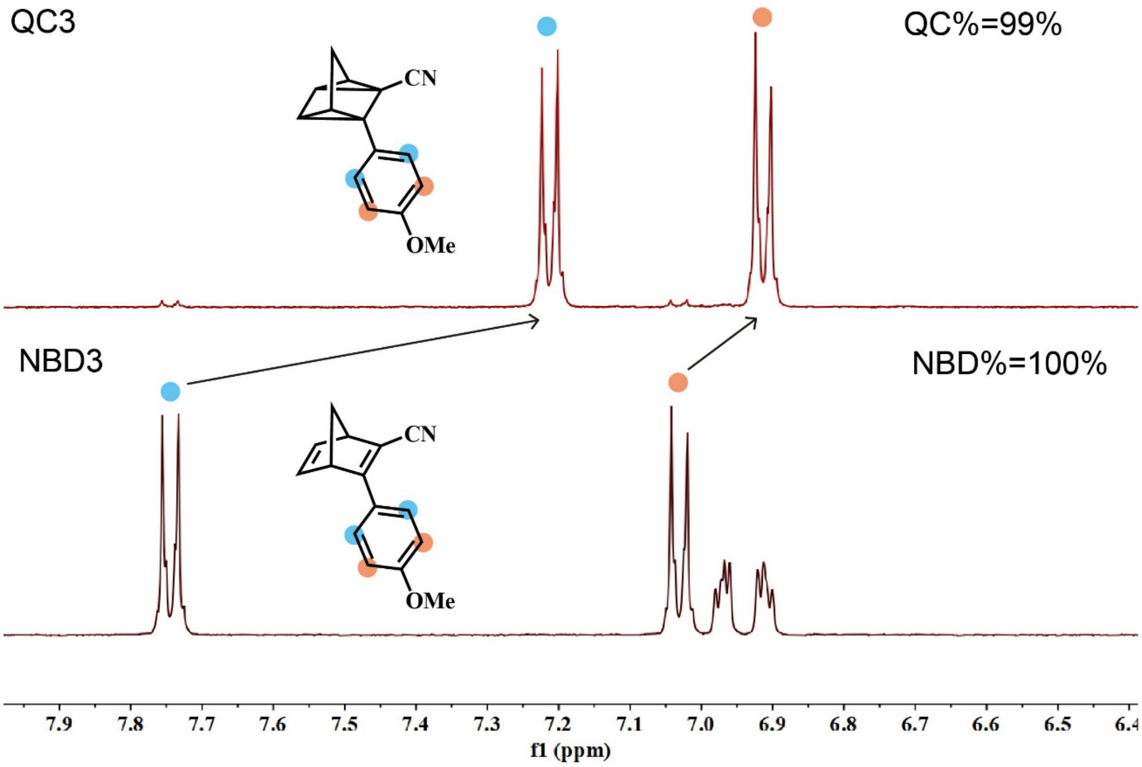

Fig. S8. ^{1}H NMR spectra and photoconversion yield of NBD3 under 365 nm light irradiation (solvent: CD_3CN).

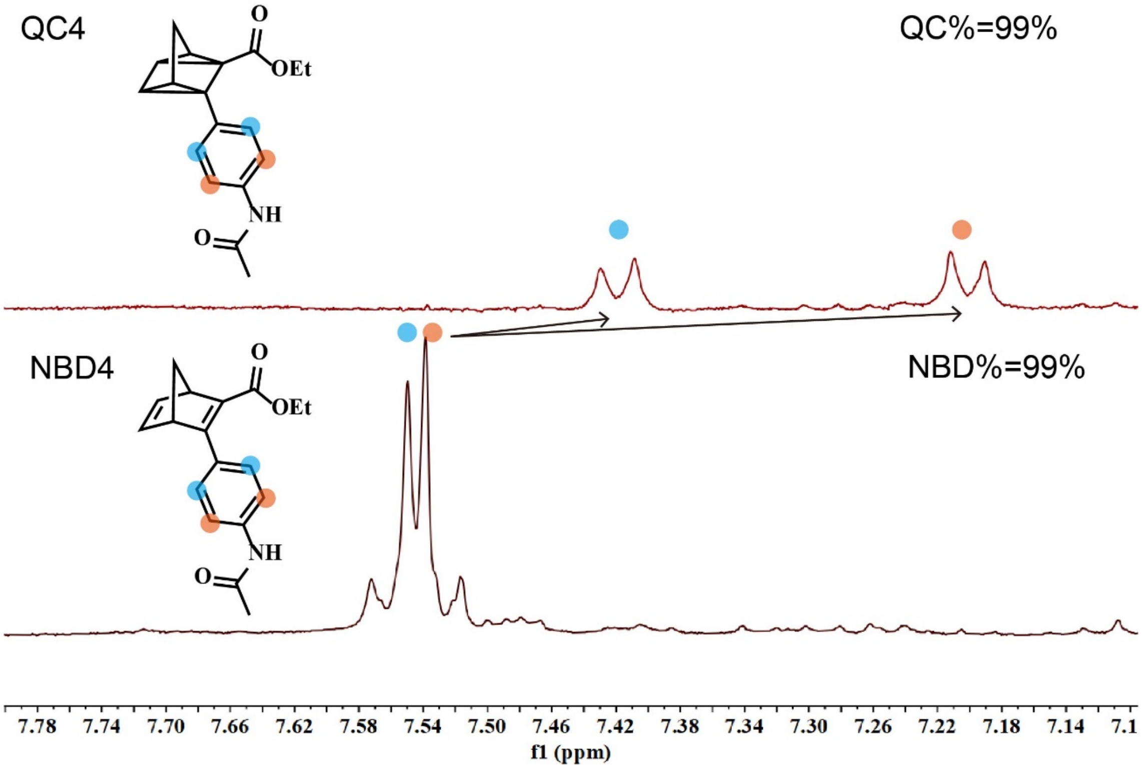

Fig. S9. ^{1}H NMR spectra and photoconversion yield of NBD4 under 365 nm light irradiation (solvent: CD_3CN).

The photoconversion yields of NBD1, NBD2, NBD3, and NBD4 were calculated after 365 nm light irradiation. These molecules converted to QC-rich photostationary states. NBD-to-QC conversions quantified using ^{1}H-NMR were 95%, 6%, 99% and 98%, respectively. The photoconversion yield of NBD2 was significantly lower than those of other NBD derivatives, owing to its fast thermal

back-conversion. The ^{1}H-NMR test was at 25 °C and need 15 min, however, QC2 was spontaneously converted back to NBD2 during the testing time.

Table S1. Comparison of photoconversion yields with reported NBD derivatives

NBD derivatives	QC%[a]	NBD%[b]	Time (min)	Irradiation wavelength (nm)	Effective conversion (%)[c]	Ref.
2-NBD	100	57	30	266	57	13
NBD/QC–fullerene hybrid system	100	15	>300	400	15	14
Fluorescent norbornadiene-based photoswitches	-	-	<1	340	<90[d]	15
NBD-based vinyl polymer	-	-	40	250	<62[d]	16
Poly(ester-amide)s containing NBD residues	100	64	30	272	64	17
NBD1	97	31		275, 265, 250	28	This study
NBD2	-	-	5	275, 265, 250	<51[d]	
NBD3	99	38	14	275, 265, 250	37	
NBD4	99	82	20	275, 265, 250	81	

[a] Photoconversion yields of NBD to QC;
[b] Photoconversion yields of QC to NBD;
[c] Effective photoconversion yields of QC to NBD, which is NBD%-(1-QC%);
[d] Photoconversion yields of NBD to QC and QC to NBD were not given, and the data were calculated from UV-Vis spectra.

2.1.5 Photoisomerization quantum yields (*Φ*) calculation

The photoisomerization quantum yield determines the efficiency of a photoreaction. The *Φ* measurement of NBD molecules in the solution is based on the reported method.[12, 18]

Before testing photoisomerization quantum yields, it is assumed that the light source is monochromatic with a collimated beam profile. The photon fluxes of the irradiation sources (365 nm and 265 nm LED lamps) were determined using potassium ferrioxalate actinometry. We used a known volume (V_1, 3 mL) of ferrioxalate solution (30 mM in 0.2 N H_2SO_4) is irradiated under stirring. 0.6 mL of irradiated solution (V_2) was mixed with 1 mL of buffer (1.2 M NaAc + 0.72 N H_2SO_4) and 2 mL of phenanthroline solution (6 mM), and then it was diluted to 25 mL (V_3) with demineralized water under dark to react over 1 h. The photon flux (I, E s^{-1}) can be calculated as following:

$$I = slope * \frac{V_1 \cdot V_3}{V_2 \cdot \varepsilon_{510\,nm} \cdot l \cdot \Phi} \qquad (S3)$$

Where l is the path length of the cuvette. $\varepsilon_{510\text{ nm}}$ corresponds to the absorbance of tri-phenanthroline complex, equal to 11100 M^{-1} cm^{-1}. And *Φ* is the photochemical quantum yield of ferrioxalate decomposition from 365 nm (1.21) and 265 nm (1.25) irradiation.[18, 19] The photonchemical quantum yields of ferrioxalate decomposition formed from 365 and 265 nm irradiation are 1.23 and 1.25, respectively. To ensure that all photons were absorbed, the concentration of the solutions were prepared to be optically thick at the irradiation wavelength. Under irradiating the sample with different time scale continuously, the absorbance changes of NBD or QC have been recorded. When all photons were absorbed, a linear dependence between the decrease in absorption and the irradiation time can be obtained as following:

$$A = A_0 - \frac{\Phi \cdot I \cdot l \cdot \varepsilon}{N_A \cdot V} \cdot t_{irr} \qquad (S4)$$

Where A and A_0 correspond to the actual and initial concentration of the NBD/QC solution, respectively. N_A, V and t_{irr} are the Avogadro constant, volume of the irradiated sample, and irradiation time, respectively.

Table S2. Experimental measurements of the photon flux under 365 nm irradiation in acetonitrile.

Photon flux of 365 nm light		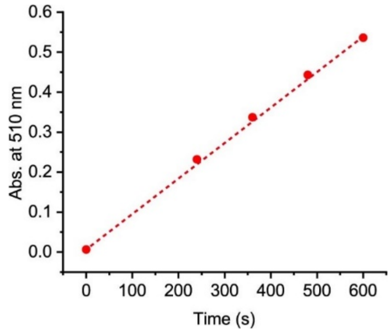
Irradiation time (s)	Tri-phenanthroline complex absorbance at 510 nm (ε = 11100 M^{-1} cm^{-1})	
0	0.006635	
240	0.2321	
360	0.3375	
480	0.4433	
600	0.5363	
Photon flux (E s^{-1})		8.121×10^{-6}

Table S3. Experimental measurements of the photon flux under 265 nm irradiation.

Photon flux of 265 nm light		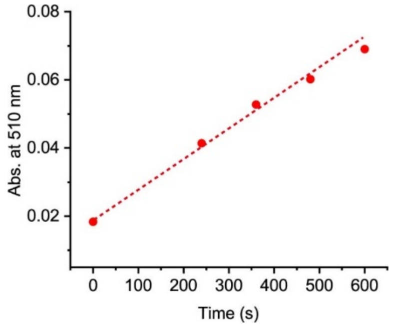
Irradiation time (s)	Tri-phenanthroline complex absorbance at 510 nm (ε = 11100 M^{-1} cm^{-1})	
0	0.01837	
240	0.04142	
360	0.05280	
480	0.06021	
600	0.06905	
Photon flux (E s^{-1})		7.450×10^{-7}

Table S4. Experimental measurements of the quantum yield of NBD1 under 365 nm irradiation in acetonitrile.

NBD1 under 365 nm irradiation			
Sample weight (g): 2.256		Sample weight (g): 2.383	
Irradiation time (s)	NBD1 abs. at 370 nm (ε = 609.6 M^{-1} cm^{-1})	Irradiation time (s)	NBD1 abs. at 370 nm (ε = 609.6 M^{-1} cm^{-1})
0	0.9041	0	0.5373
20	0.8765	10	0.5229
40	0.8477	20	0.5125
60	0.8213	30	0.4964
80	0.8033	40	0.4837
Quantum yield: 58%	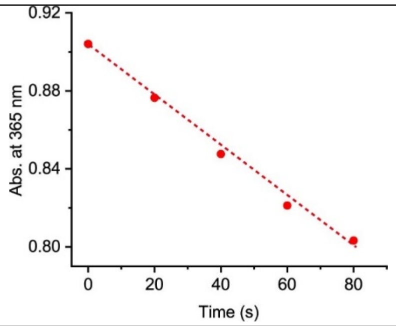	Quantum yield:64%	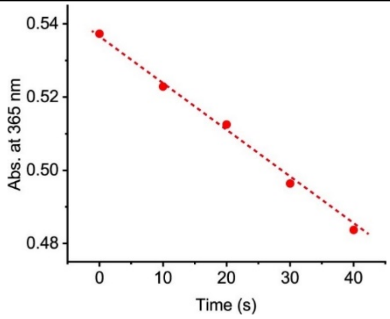
Quantum yield: 62%			

Table S5. Experimental measurements of the quantum yield of NBD2 under 365 nm irradiation in acetonitrile.

NBD2 under 365 nm irradiation			
Sample weight (g): 2.453		Sample weight (g): 2.414	
Irradiation time (s)	NBD2 abs. at 435 nm (ε = 1161 M^{-1} cm^{-1})	Irradiation time (s)	NBD2 abs. at 435 nm (ε = 1161 M^{-1} cm^{-1})
0	0.8444	0	0.5826
20	0.8154	20	0.5607
40	0.7989	40	0.5362
60	0.7730	60	0.5168
80	0.7549	80	0.4990
Quantum yield: 29%	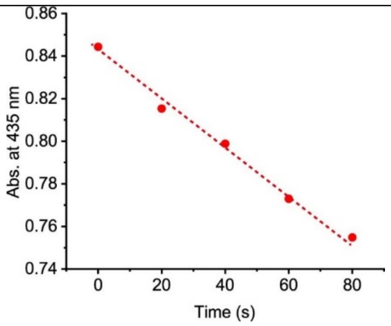	Quantum yield: 27%	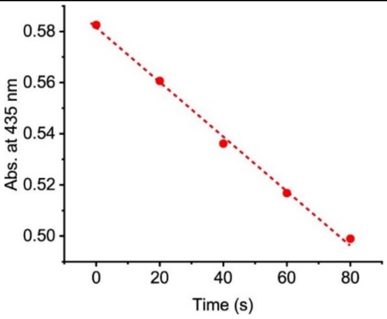
Quantum yield: 28%			

Table S6. Experimental measurements of the quantum yield of NBD3 under 365 nm irradiation in acetonitrile.

NBD3 under 365 nm irradiation			
Sample weight (g): 2.472		Sample weight (g): 2.624	
Irradiation time (s)	NBD3 abs. at 370 nm (ε = 524.3 M^{-1} cm^{-1})	Irradiation time (s)	NBD3 abs. at 370 nm (ε = 524.3 M^{-1} cm^{-1})
0	0.9484	0	0.3468
20	0.9362	20	0.3358
40	0.9181	40	0.3237
60	0.9103	60	0.3131
80	0.8952	80	0.3020
Quantum yield: 38%	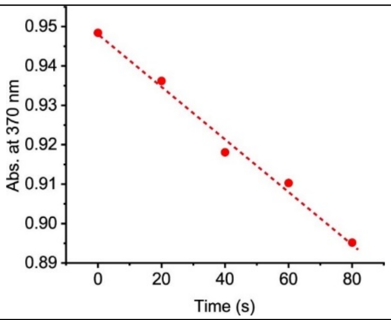	Quantum yield:35%	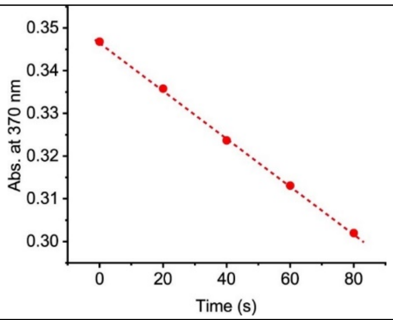
Quantum yield: 37%			

Table S7. Experimental measurements of the quantum yield of NBD4 under 365 nm irradiation in acetonitrile.

NBD4 under 365 nm irradiation			
Sample weight (g): 2.219		Sample weight (g): 2.568	
Irradiation time (s)	NBD4 abs. at 350 nm (ε = 2192 M^{-1} cm^{-1})	Irradiation time (s)	NBD4 abs. at 350 nm (ε = 2192 M^{-1} cm^{-1})
0	1.068	0	0.8974
10	0.9849	5	0.8692
20	0.9361	10	0.8474
30	0.8801	15	0.8150
40	0.8185	20	0.7968
Quantum yield: 75%	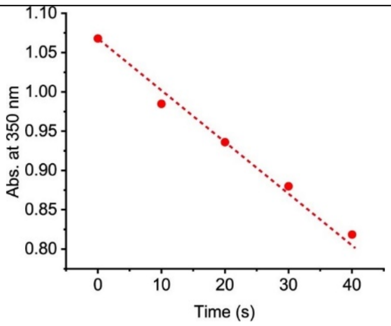	Quantum yield:74%	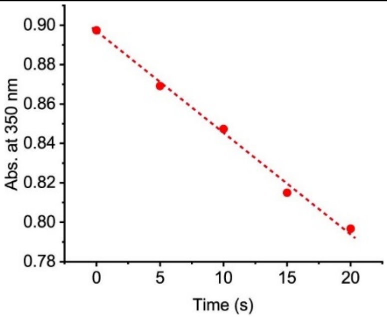
Quantum yield: 75%			

Table S8. Experimental measurements of the quantum yield of QC1 under 265 nm irradiation in acetonitrile.

QC1 under 265 nm irradiation			
Sample weight (g): 2.156		Sample weight (g): 2.082	
Irradiation time (s)	QC1 abs. at 290 nm (ε = 8352 M^{-1} cm^{-1})	Irradiation time (s)	QC1 abs. at 290 nm (ε = 8352 M^{-1} cm^{-1})
0	0.9035	0	0.9814
10	0.9080	10	0.9887
20	0.9128	20	0.9896
30	0.9166	30	0.9942
40	0.9201	40	0.9988
Quantum yield: 28%	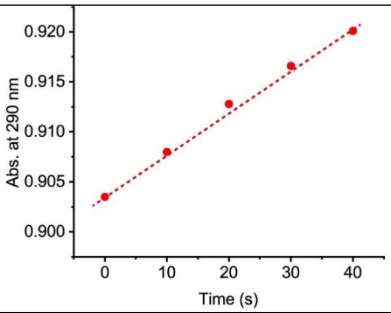	Quantum yield: 24%	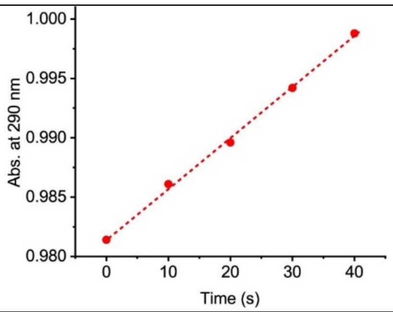
Quantum yield: 26%			

Table S9. Experimental measurements of the quantum yield of QC3 under 265 nm irradiation in acetonitrile.

QC3 under 265 nm irradiation			
Sample weight (g): 2.023		Sample weight (g): 2.156	
Irradiation time (s)	QC3 abs. at 300 nm (ε = 7722 M^{-1} cm^{-1})	Irradiation time (s)	QC3 abs. at 300 nm (ε = 7722 M^{-1} cm^{-1})
0	0.7302	0	0.7638
10	0.7431	10	0.7748
20	0.7532	20	0.7839
30	0.7645	30	0.7941
40	0.7751	40	0.8060
Quantum yield: 38%	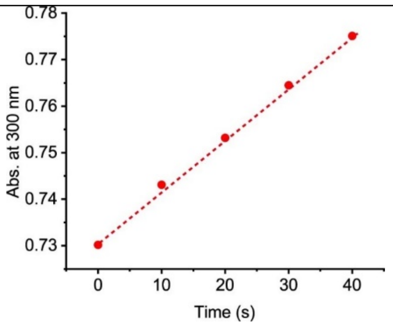	Quantum yield: 39%	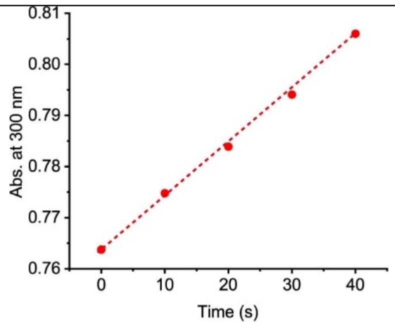
Quantum yield: 39%			

Table S10. Experimental measurements of the quantum yield of NBD4 under 265 nm irradiation in acetonitrile.

QC4 under 265 nm irradiation			
Sample weight (g): 2.073		Sample weight (g): 2.393	
Irradiation time (s)	QC4 abs. at 315 nm (ε = 6548 M^{-1} cm^{-1})	Irradiation time (s)	QC4 abs. at 315 nm (ε = 6548 M^{-1} cm^{-1})
0	0.9365	0	0.7692
15	0.9618	15	0.7877
30	0.9809	30	0.8012
45	1.002	45	0.8192
60	1.018	60	0.8347
Quantum yield: 54%	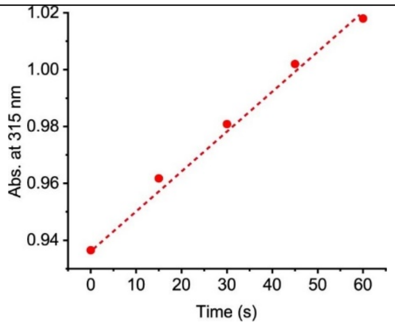	Quantum yield: 52%	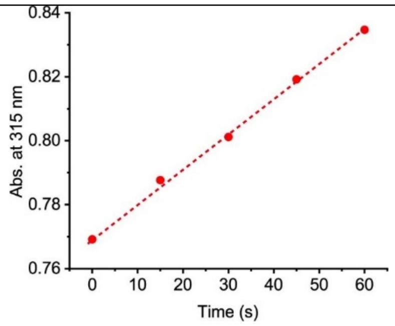
Quantum yield: 53%			

2.1.6 Thermal back conversion

The acetonitrile solutions of NBD derivatives were irradiated to obtain QC until no further change was observed in the absorbance profile. And then the increase of the temperatures over time was measured by monitoring the absorption. All the curves are fitted by first-order kinetics equation as following:

$$y = -Ae^{-k} + y_0 \quad (S5)$$

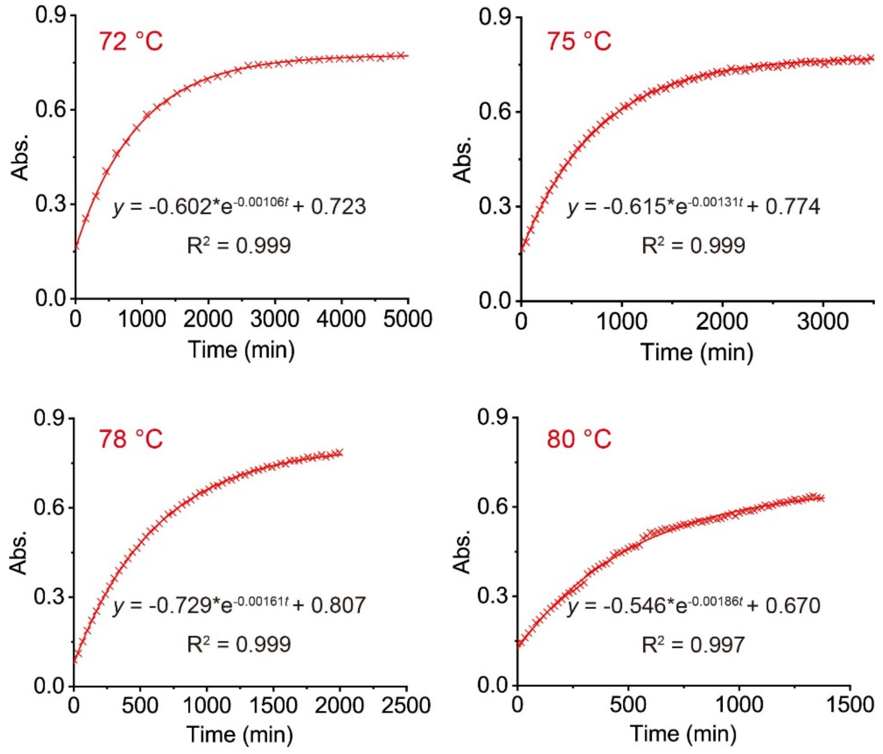

Fig. S10. Thermal back conversion of NBD1@315 nm at 72, 75, 78, 80 °C in MeCN solution (0.1 mM L^{-1}).

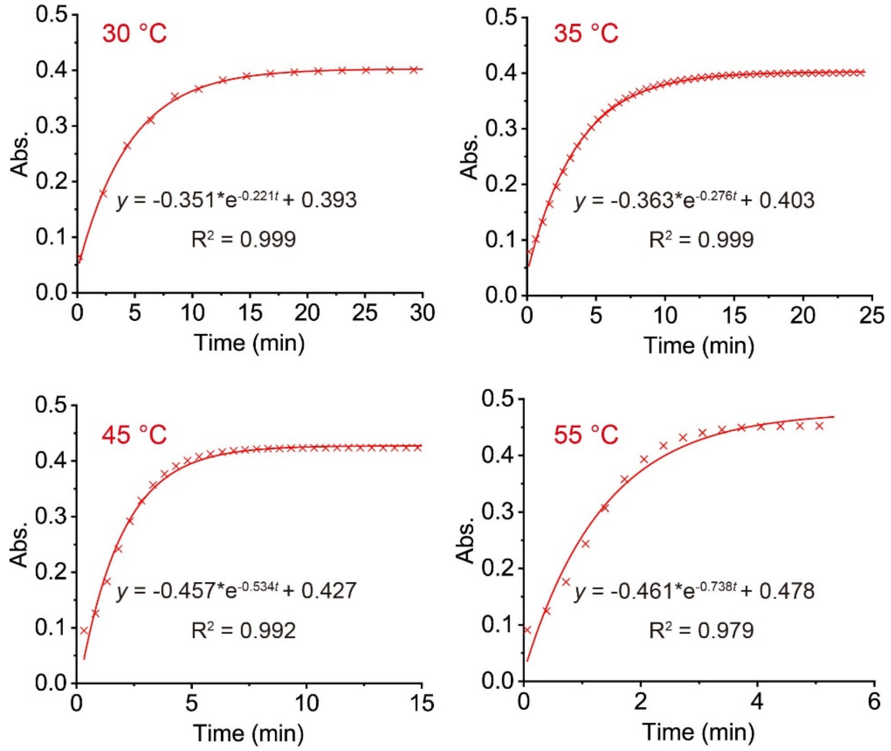

Fig. S11. Thermal back conversion of NBD2@315 nm at 30, 35, 45, 55 °C in MeCN solution (0.1 mM L^{-1}).

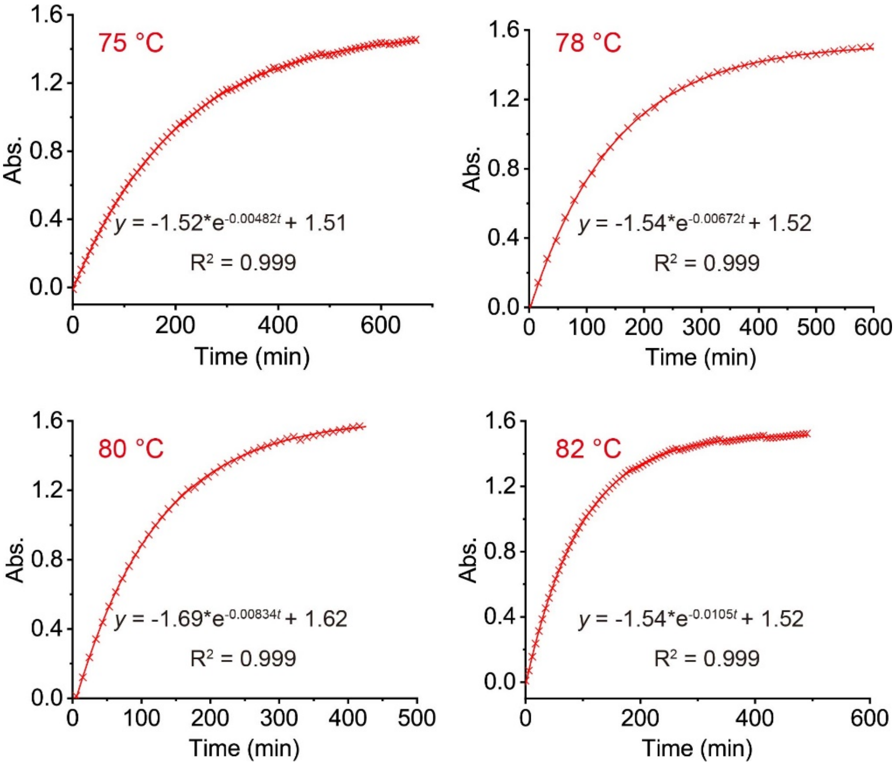

Fig. S12. Thermal back conversion of NBD3@315 nm at 75, 78, 80, 82 °C in MeCN solution (0.1 mM L^{-1}).

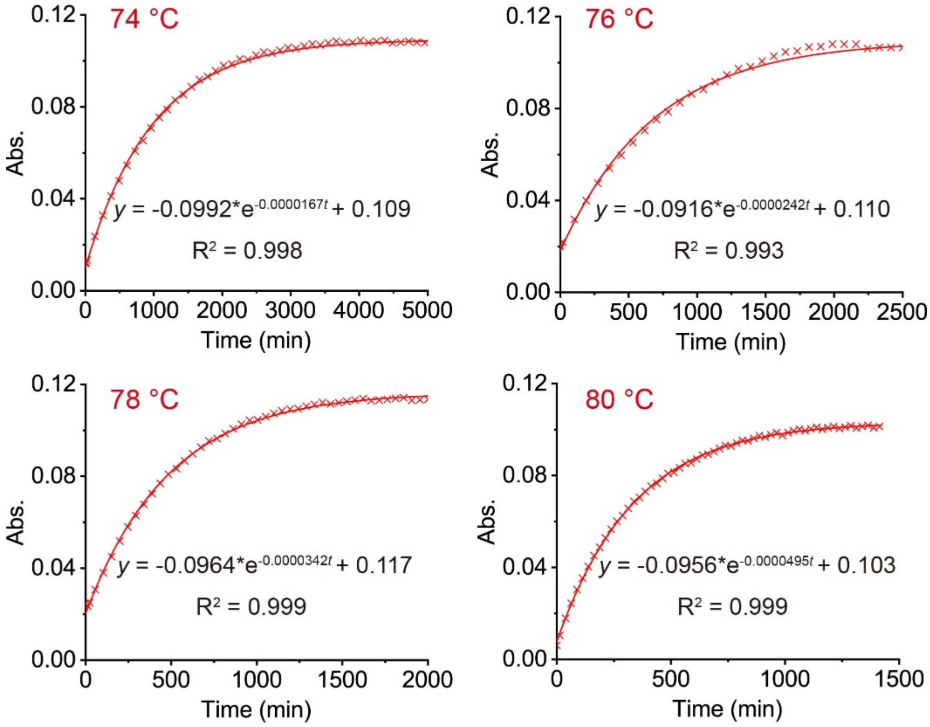

Fig. S13. Thermal back conversion of NBD4@365 nm at 74, 76, 78, 80 °C in MeCN solution (0.1 mM L^{-1}).

The measurements were performed at four different temperatures (Fig. S7-10) and an exponential fit of the Arrhenius equation and Eyring equation was applied to the data to determine the apparent activation energy (E_a), enthalpy ($\Delta H^‡$) and entropy ($\Delta S^‡$) of activation.

Arrhenius equation

$$lnk = \frac{-E_a}{RT} + C \qquad (S6)$$

where

k = reaction rate constant

T =absolute temperature

R= gas constant

E_a = apparent activation energy

Eyring equation

$$ln\frac{k}{T}=\frac{-\Delta H^{\ddagger}}{R}\cdot\frac{1}{T}+ln\frac{k_B}{h}+\frac{\Delta S^{\ddagger}}{R} \qquad (S7)$$

where:

k = reaction rate constant

T =absolute temperature

R= gas constant

k_B = Boltzmann constant

h = Planck' s constant

$\Delta H^{\ddagger}$ = Enthalpy of activation

$\Delta S^{\ddagger}$ = Entropy of activation

Table S11. Thermodynamic parameters of NBDs (calculated from **Fig. S14**)

Samples	$\Delta H^{\ddagger}$ (kJ mol^{-1})	$\Delta S^{\ddagger}$ (J mol^{-1} K^{-1})
NBD1	67.6	-141.2
NBD2	38.9	-163.1
NBD3	106.2	-10.6
NBD4	177.2	-4.01

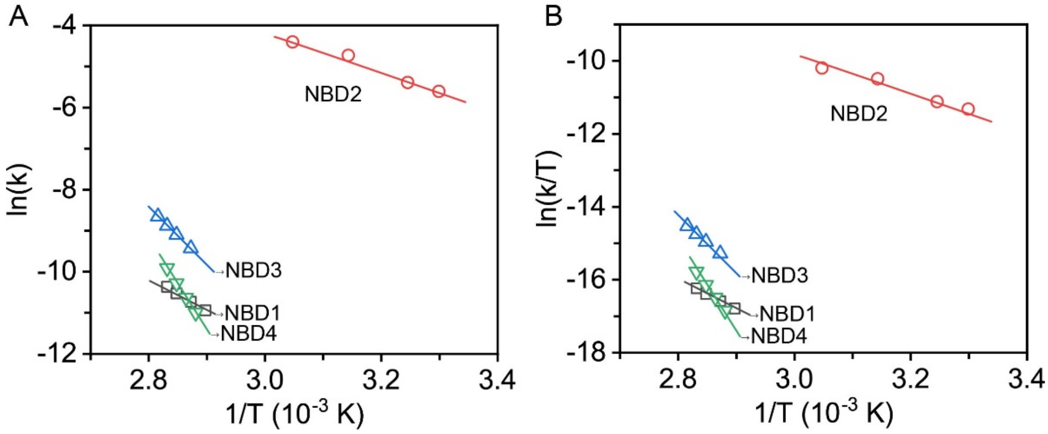

Fig. S14. (A) Arrhenius plot of NBD derivatives. (B) Eyring plot of NBD derivatives.

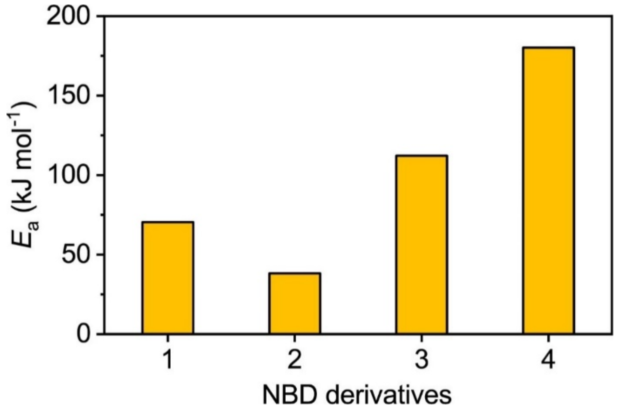

Fig. S15. Apparent activation energy of NBD derivatives.

2.2 Photostability and Photodegradation

2.2.1 Photodegradation of NBD derivatives in ^1H NMR spectra

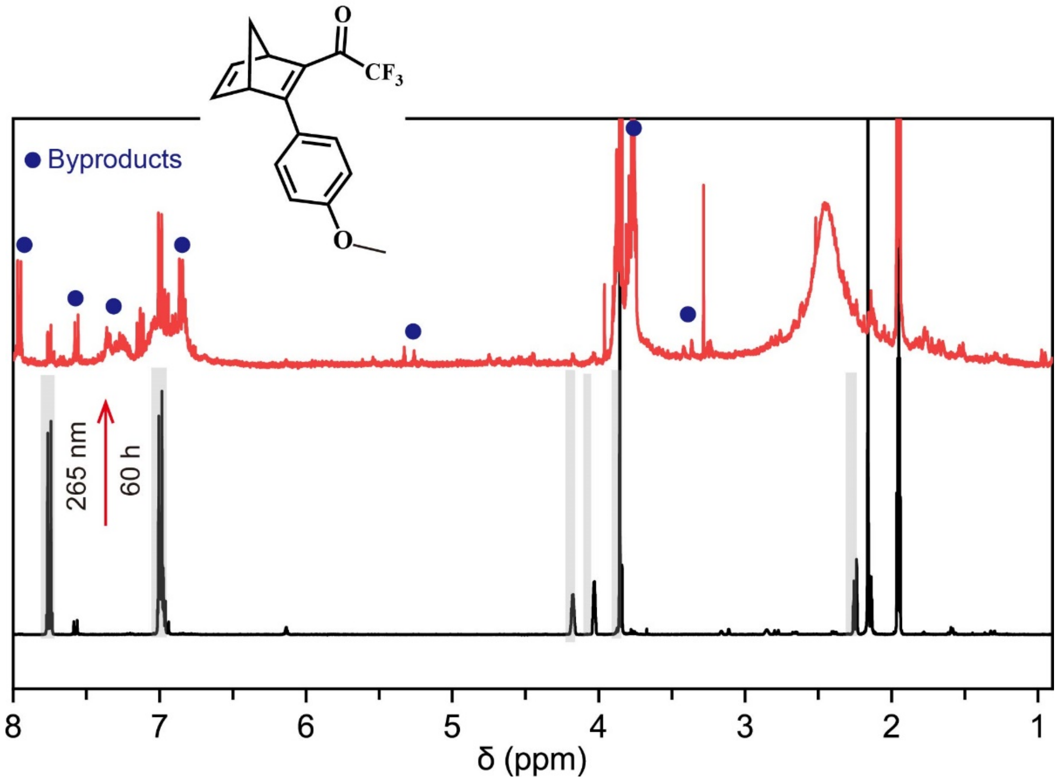

Fig. S16. ^1H NMR spectra of NBD2 before and after 265 nm light irradiation. The vertical axis of NBD2 after 265 nm light irradiation is magnified 10 times.

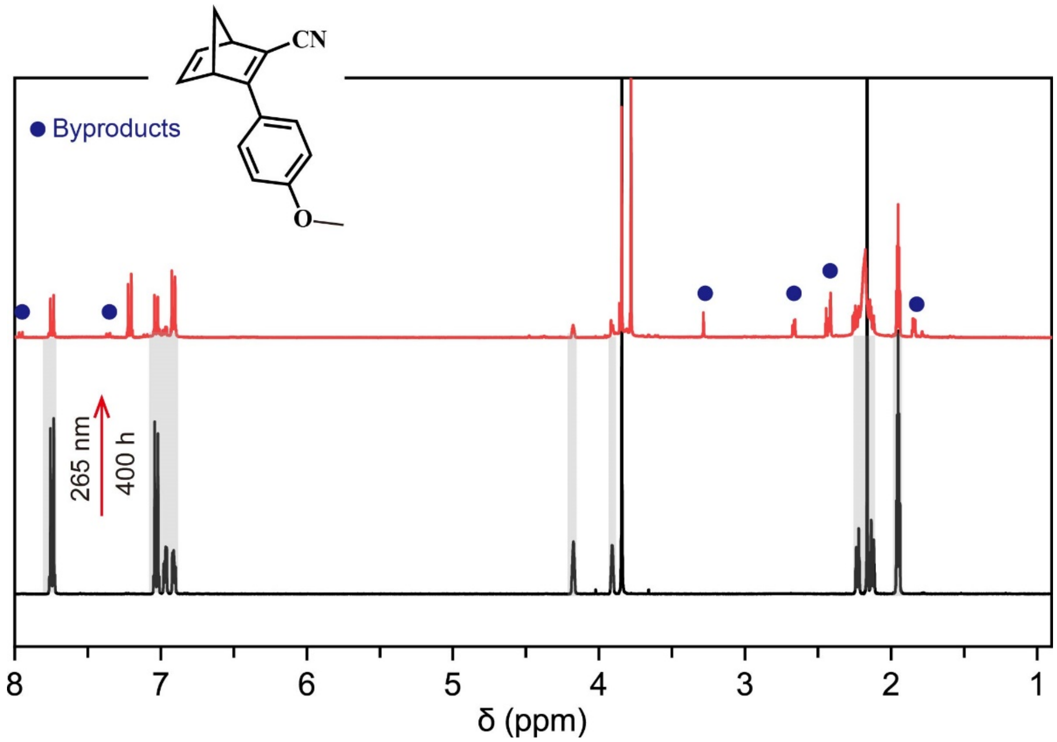

Fig. S17. ^1H NMR spectra of NBD3 before and after 265 nm light irradiation.

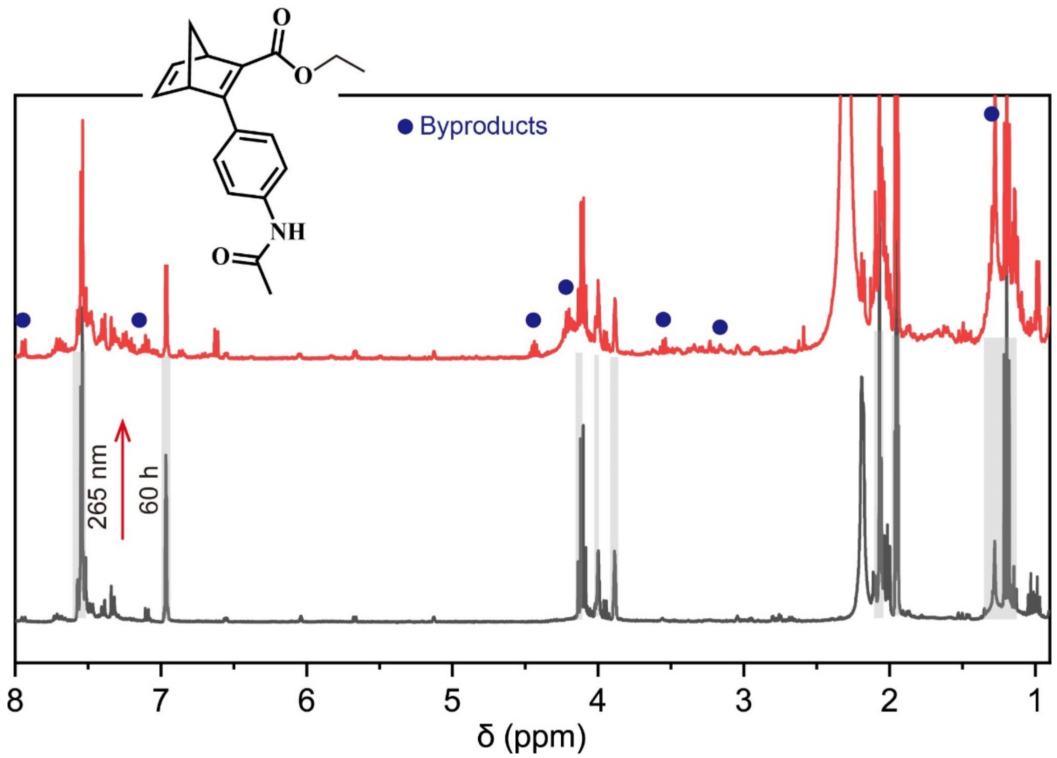

Fig. S18. ¹H NMR spectra of NBD4 before and after 265 nm light irradiation. The vertical axis of NBD4 after 265 nm light irradiation is magnified 4 times

2.2.2 Photodegradation of NBD derivatives in HRMS

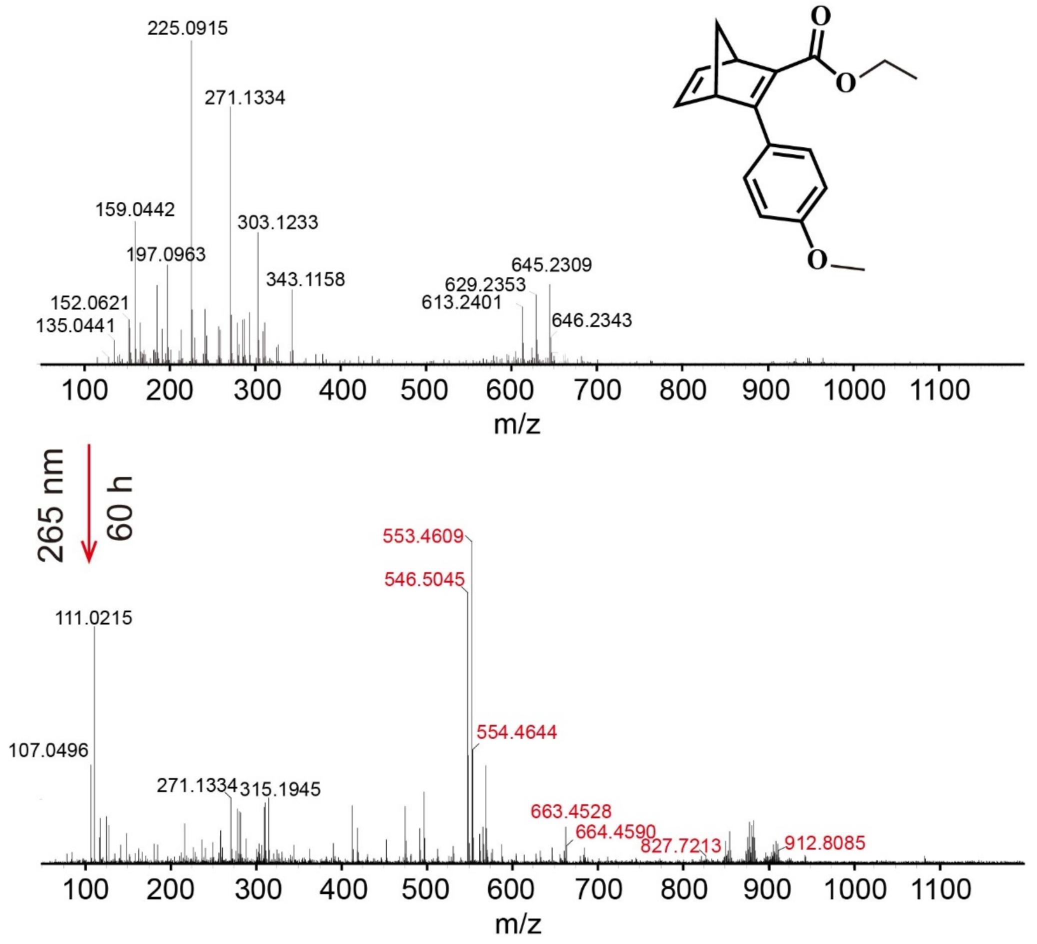

Fig. S19. HRMS spectra of NBD1 before and after 265 nm light irradiation.

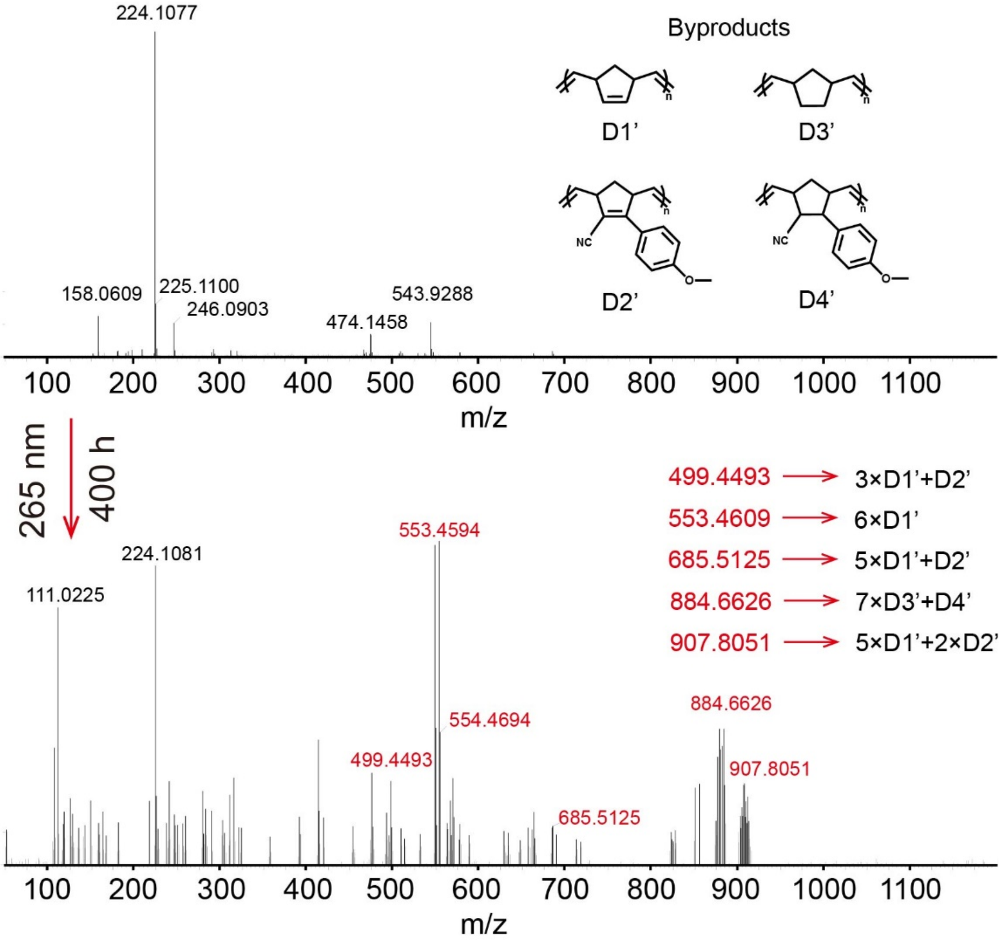

Fig. S20. HRMS spectra of NBD3 before and after 265 nm light irradiation.

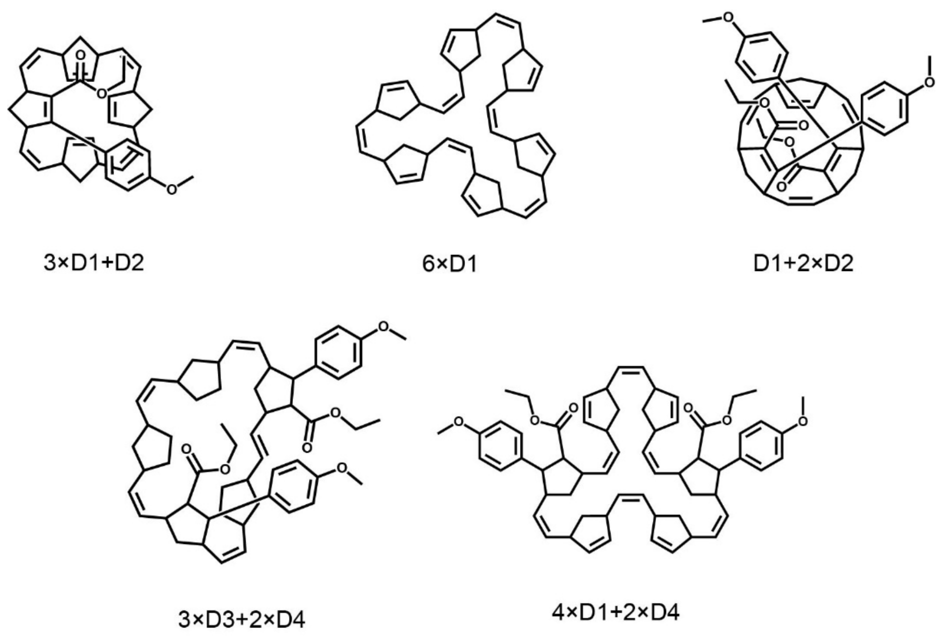

Fig. S21. Byproducts of NBD1 after 265 nm light irradiation.

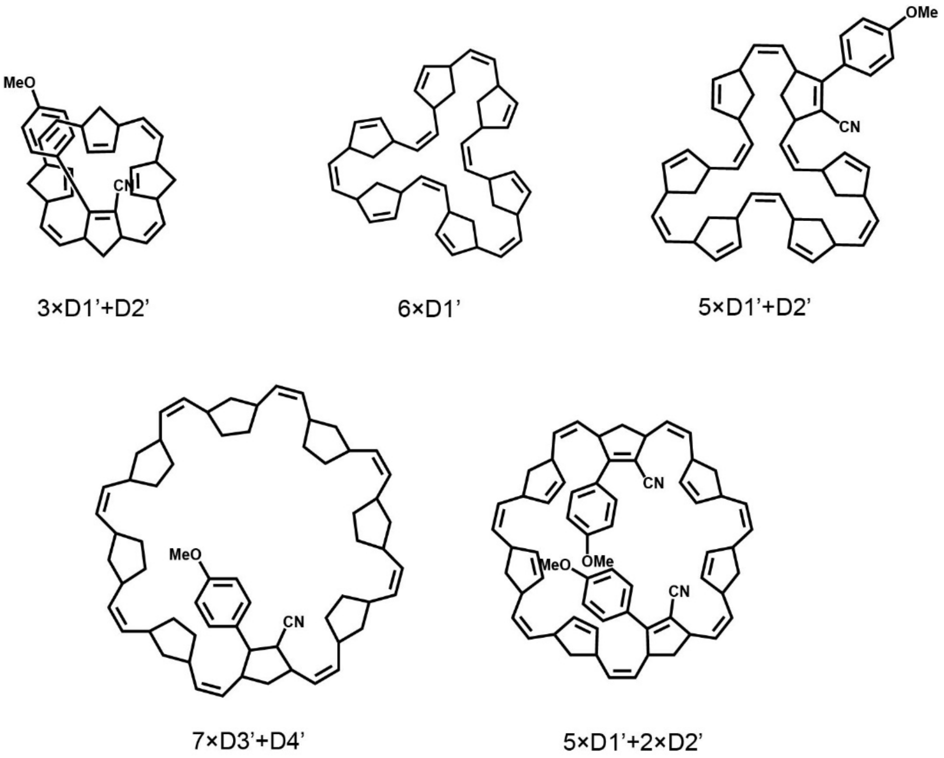

Fig. S22. Byproducts of NBD3 after 265 nm light irradiation.

2.2.3 Photodegradation comparison of optical and thermal back-conversion

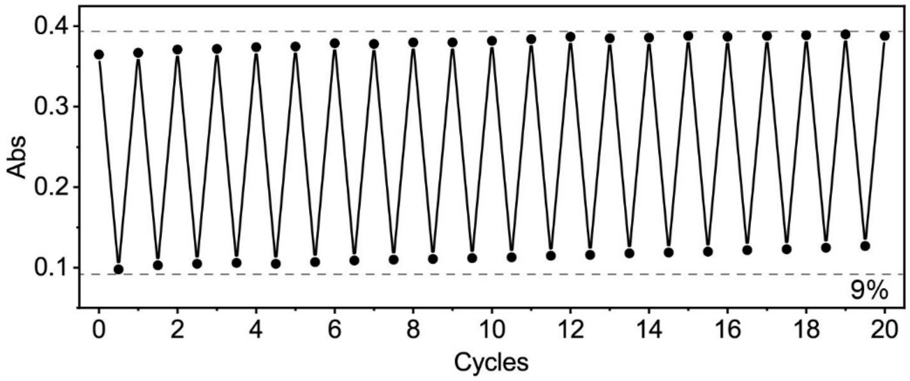

Fig. S23. Cycling test of NBD4 in acetonitrile. The NBD-to-QC conversion was under 365 nm light irradiation, and the QC-to-NBD conversion was accelerated by heating (80 °C). The photodegradation yield of NBD4 via thermoback conversion was significantly lower than that under 265 nm light irradiation, showing that the short UV wavelength irradiation could lead to a serious photodegradation.

2.2.4 UV-Vis absorption spectra after photodegradation

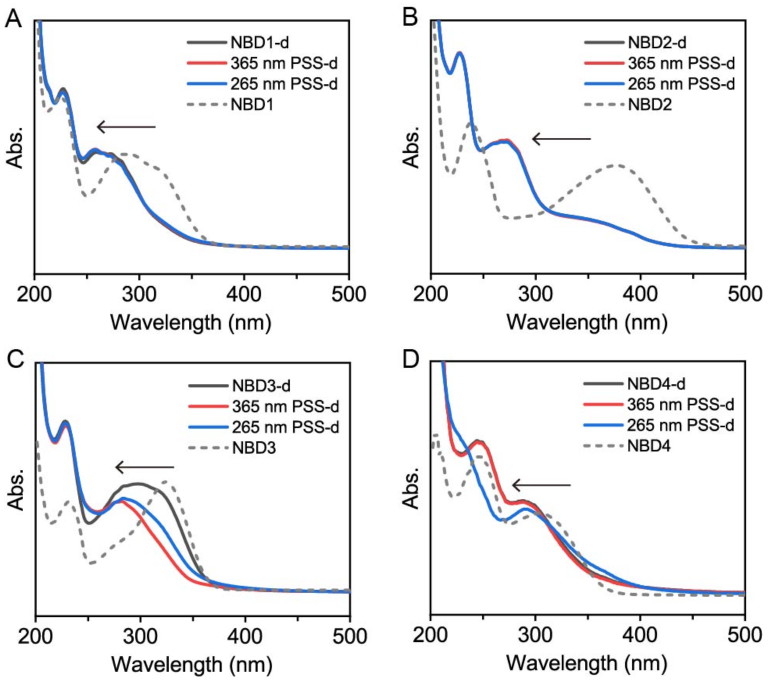

Fig. S24. UV-Vis absorption spectra in MeCN (0.1 mM L^{-1}) of (A) NBD1, (B) NBD2, (C) NBD3 and (D) NBD4 after 265 nm light irradiation.

2.3 Solar Energy Storage and Release by Light

Various photoswitchable molecules have been applied to store the solar energy, including NBD/QC couple, azobenzene derivatives, and the dihydroazulene/vinylheptafulvene (DHA/VHF) couple. However, only azobenzene derivatives presented the energy release by photoswitching in previous works. In this study, we reported an optical energy release approach in NBD/QC couple. A comparison of efficient energy storage density in two-way photoswitching system was presented in Fig. 3B.

Table S12. The energy storage capacity of NBDs.

Samples	ΔH_S (J g^{-1})	ΔH_R (J g^{-1})	ΔH_E (J g^{-1})
NBD1	325	244	81
NBD2	212	105	107
NBD3	406	290	116
NBD4	395	83	312

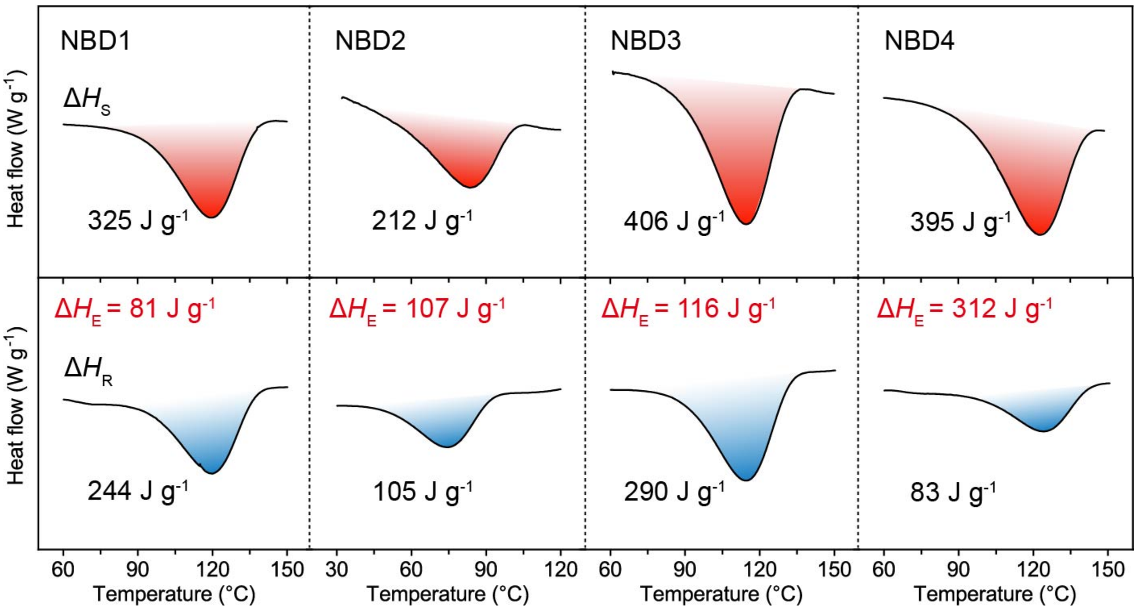

Fig. S25. DSC thermograms for energy release of NBDs at 365 nm PSS and 265 nm PSS.

2.4 Automation Flow MOST Device

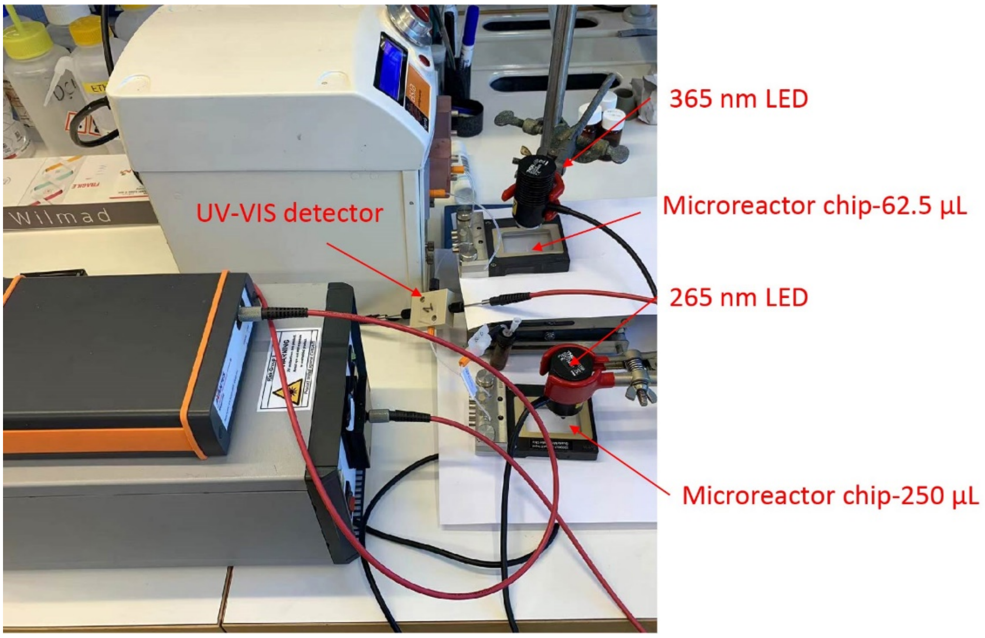

Fig. S26. Optical image of the automation flow MOST device.

3. Cartesian Coordinates

NBD1

C	3.68280713381003	2.24565251297529	1.82305248423813
C	3.82492998495444	1.99530501064408	0.33487837988499
O	3.21370152407402	0.75276390319776	-0.04054137952514
C	1.88658381743157	0.74651632379533	-0.23109453682411
O	1.20930764502665	1.74909768694384	-0.13174794697574
C	1.40493474017285	-0.60761545633876	-0.53806007127198
C	0.14018449049107	-1.09274107212634	-0.45993261975447
C	-1.16004861905429	-0.45492297878239	-0.24391321073195
C	-2.13391366642956	-1.12517034653237	0.52363137141102
C	-3.38066761010208	-0.56548861393392	0.74962885290776
C	-3.70484851210958	0.67733623513111	0.18373573944329
O	-4.93959670614610	1.15554421760518	0.44810933245298
C	-5.36483600590877	2.32949012121057	-0.22198175895110
C	-2.76127739166163	1.34717383500545	-0.60580601945935
C	-1.50326146086235	0.78164808319686	-0.80602120287682
C	0.26957313778461	-2.62760401291077	-0.55369564158232
C	1.01278359022417	-3.08740682550405	0.71195000699763
C	2.25680536968692	-2.60504294470829	0.63232889222241
C	2.34584287150708	-1.81717745289341	-0.68574377333651
C	1.43785451554677	-2.70650522362819	-1.56644408970561
H	2.62649354029156	2.34148909938415	2.10056797075354
H	4.13158352900213	1.42245839106248	2.39233222794744
H	4.19876178997297	3.17688368681879	2.08747732646904
H	4.87550235592326	1.88646077887175	0.04841123012070
H	3.37469252312653	2.80323853553568	-0.25268944372309
H	-1.90165774280263	-2.09587620532700	0.96267140360253
H	-4.12631963896411	-1.07762491544246	1.35681752881296
H	-5.27359985602376	2.20804056989930	-1.31043467757773
H	-4.77465758649752	3.19848293750001	0.10195321938971
H	-6.41295239917275	2.47525236070702	0.04745426938409
H	-2.99645721219617	2.30325387647214	-1.06755421959723
H	-0.77678793093356	1.30442255273091	-1.42277794864517
H	-0.66045886772940	-3.15197882265663	-0.78885743529380
H	0.55405140686244	-3.64424284110256	1.52639674724341
H	3.05485044626881	-2.67874821155975	1.36822759699751

H	3.34947019641751	-1.58285219569334	-1.04533048639923
H	1.81940991730863	-3.72573235821138	-1.69151747174948
H	1.20117668071024	-2.25380024133611	-2.53648064629832

QC1

C	4.27456905014342	1.93707626857209	0.87377943807114
C	4.00570027728390	1.60375604569619	-0.57982931195726
O	3.28030259670708	0.37073945553444	-0.70396765345900
C	1.95600611125601	0.41006548796174	-0.51046860351993
O	1.35161327456818	1.43759718777482	-0.28850360466434
C	1.35878266280616	-0.92760411799241	-0.57752839330302
C	-0.07146315981946	-1.38260392303586	-0.19879623267663
C	-1.24959192438436	-0.54815221845642	0.12787869404419
C	-1.58876358227777	0.55567691855702	-0.65954494838402
C	-2.72086915288166	1.32582140873189	-0.38859090212202
C	-3.54587340556241	0.98554246973512	0.69008992716364
O	-4.66426205234319	1.67225651701769	1.03351227524423
C	-5.00455121695653	2.81657725279186	0.27475478455563
C	-3.22430962971364	-0.12446521862830	1.48198617500716
C	-2.08827943131034	-0.87460158295152	1.20368231430858
C	-0.17325551406066	-2.76654048285313	-0.81769460516085
C	0.49851347661150	-2.58483450133164	0.51824019950984
C	1.92763373457211	-2.14455093147939	0.15336333503656
C	1.96500579656054	-2.09969512333198	-1.33716871622169
C	0.84928754470333	-2.94069148010537	-1.91943867492160
H	4.80584302275578	1.11007886751174	1.36083232276149
H	4.89942226970534	2.83675448488327	0.93221990563588
H	3.33683086639582	2.12783694974962	1.40875070767277
H	4.93725252951555	1.43353210520910	-1.12802741003690
H	3.43833680575958	2.39866003903981	-1.07691427772896
H	-0.95052074652327	0.82774271287799	-1.49944029681963
H	-2.94979269136116	2.18045587036611	-1.02119310564468
H	-4.19595086118653	3.56106566913189	0.30691759757387
H	-5.90586927566987	3.23266215720210	0.73016217245289
H	-5.20967293939104	2.54492633664994	-0.77089151943405
H	-3.87388777739145	-0.37881531538849	2.31925459104074
H	-1.84399931566026	-1.72792070279136	1.83699298888145
H	-1.16050210527265	-3.22230454897994	-0.85785291197118

H	0.17666324148401	-3.04166240169685	1.45024925177146
H	2.79898039285745	-2.12972951188733	0.80158661444583
H	2.91067660944947	-1.93875620307061	-1.84845077236674
H	0.49447863737501	-2.54833311788443	-2.88142781866725
H	1.15548588125604	-3.98757282312942	-2.04583353611759

TS1

C	4.42673115463964	1.49827444798963	0.66424887187938
C	3.94431140758240	1.66252312811123	-0.76599997388104
O	3.14071806161123	0.56090156355175	-1.17360814409227
C	1.82739265793952	0.54461323777669	-0.75278890710256
O	1.35336972147793	1.54316780361673	-0.19482284051499
C	1.19015134131626	-0.67196373158428	-1.08008626944380
C	-0.05358099317019	-1.21777448045917	-0.41304782277051
C	-1.23934920691298	-0.46773094786629	0.00846496836448
C	-1.47428067366481	0.81123583702426	-0.51020639859717
C	-2.66028943820798	1.49443679718569	-0.24034066929189
C	-3.62488070216116	0.90620133511010	0.58457790743400
O	-4.79129973141047	1.49124848744979	0.91654936926352
C	-5.07259464681190	2.78054147102415	0.39770623638534
C	-3.39694430664598	-0.37555399126966	1.11997308626949
C	-2.23117902709022	-1.05315365091230	0.82293081381797
C	-0.11317776646579	-2.68601138563854	-0.60813423605610
C	0.58834770008542	-2.44038769830598	0.72467126399090
C	1.87746055269784	-2.03645482810456	0.36444430302902
C	1.99937847281823	-1.99047204908566	-1.11868493232295
C	0.99299075694215	-3.04113232387176	-1.59483248573502
H	4.99816825790882	0.56678554131285	0.76754057196376
H	5.07608954359447	2.33841533038187	0.94137148455956
H	3.57443899509995	1.47248322949733	1.35414731420912
H	4.79069753440968	1.67273548493198	-1.46228406862221
H	3.38209875502652	2.59573451067995	-0.88656985072735
H	-0.71899728850083	1.27888773735311	-1.13499226347591
H	-2.81488472138778	2.48059836104389	-0.67103648623347
H	-4.31970941375033	3.50620486111887	0.73578789048235
H	-6.05511381684691	3.05888888415517	0.78364226560877
H	-5.09614041030778	2.75823124459681	-0.70079994200813
H	-4.15704293592613	-0.81811491133751	1.76260444432678

H	-2.08298577448544	-2.04844398392373	1.24124887889553
H	-1.08173776762527	-3.18175953083835	-0.60715692479393
H	0.15381728418515	-2.45311386808914	1.71683346438637
H	2.63499797525640	-1.64564650655454	1.04577046948419
H	2.99593273661712	-1.94892548995425	-1.55219863789656
H	0.69000261519831	-2.87581315293181	-2.63445676049538
H	1.34706309696488	-4.06967676318432	-1.45777599028929

NBD2

C	5.48376058600184	-1.15866491815501	-0.08246273274308
O	4.78827530790261	0.01883270626953	0.29347431487383
C	3.45189822154119	0.05310049542900	0.14113493574550
C	2.69519813153060	-1.01764402393625	-0.35610986080417
C	1.31356830733360	-0.89187962166907	-0.46141773456897
C	0.65665787843874	0.29385111690385	-0.09911697688227
C	-0.78214949998751	0.47401816468587	-0.24044127317203
C	-1.84448935192140	-0.37451647321749	-0.07566404804542
C	-1.83038687443993	-1.70978056119163	0.48661684326012
O	-0.86863806686356	-2.39609751402265	0.76210594260695
C	-3.22936177695001	-2.32319041193739	0.78536965857108
F	-3.11950608505003	-3.50161998647565	1.38274561093767
F	-3.91993744456251	-2.50386280427511	-0.34738542402416
F	-3.95514556362970	-1.52883231194612	1.58046798695499
C	-3.11850884733223	0.46509375496593	-0.32979679787373
C	-3.19267886885382	1.49870140605430	0.80490378891689
C	-2.15169445978555	2.32170327009396	0.65046920091451
C	-1.38076106834924	1.84662423285029	-0.59105319943032
C	-2.57161266501561	1.36829522313843	-1.45780304415915
C	1.44118226528330	1.37121374810635	0.36647770170769
C	2.81199388075863	1.25054235374751	0.50387109744499
H	5.33272880118675	-1.37260906067683	-1.14960532228008
H	6.54054993849937	-0.96482906844741	0.11067687810379
H	5.14598011691293	-2.01510653628189	0.51753254567733
H	3.17183332493932	-1.94532603933735	-0.66301542825249
H	0.73574284274516	-1.72405041599726	-0.85479951727214
H	-4.03296956188216	-0.09547483103021	-0.53154548629522
H	-3.92995529804337	1.48927456906216	1.60472869128682
H	-1.83296965921652	3.13949815874794	1.29320937264849

H	-0.67213652149062	2.55402263353574	-1.02844955725345
H	-3.24976995639893	2.18602092020833	-1.72351525569432
H	-2.26440379264862	0.80678221883447	-2.34774342222188
H	0.96209387650799	2.31118966490788	0.64092463171324
H	3.41559188283930	2.07473994105577	0.88186587960896

QC2

C	5.41147469751823	0.81452015927057	-0.47683450279933
O	4.63762083252425	-0.18268966319584	-1.11644489363014
C	3.32083319143102	-0.26805066423913	-0.80581562467927
C	2.68601138505826	0.55222185934035	0.13570231586727
C	1.32399992199423	0.38364071629557	0.38542524441054
C	0.57088904935503	-0.58045236497438	-0.29038583071687
C	-0.86584951816612	-0.76307121514565	0.01775540337804
C	-1.96241687395503	0.32349284314447	0.07564468701503
C	-1.86381222330576	1.69854597439004	-0.36262375894480
O	-0.87701859235336	2.23689809554207	-0.81048454897347
C	-3.14318876378548	2.57341565858097	-0.21709555791905
F	-4.24432164028085	1.93812193416740	-0.62996424220936
F	-3.03434146503505	3.69540178683556	-0.91459869617942
F	-3.32637831560954	2.90459151955254	1.06655748417798
C	-3.08263828371302	-0.28241048319966	0.92905003270864
C	-2.97727521284798	-0.70818941535621	-0.48829011071314
C	-1.86099078567987	-1.76009929085972	-0.53019409982661
C	-1.41329890902954	-1.86426759959453	0.90447285198488
C	-2.47226813886696	-1.32487130210220	1.84134686469267
C	1.22059522241160	-1.38939160056115	-1.23252344057065
C	2.57959393416966	-1.24351774617376	-1.48508035043317
H	6.42292123474443	0.72633089013815	-0.87923177357895
H	5.43086384942284	0.65629282301888	0.61109513126491
H	5.01237635116139	1.81593282312013	-0.69349558533830
H	3.23847452386509	1.31754619644440	0.67594016522530
H	0.83586086708485	1.02422928901252	1.12059967240849
H	-3.90376012130229	0.35600139879915	1.24582485130701
H	-3.70727330594389	-0.49656335627277	-1.26300216040866
H	-1.71421329171024	-2.51484437397540	-1.29745939404235
H	-0.72800756954574	-2.65861070237333	1.19137349064280
H	-2.04163252089826	-0.88001777096956	2.74711117404225

H	-3.21153974309661	-2.08512103986622	2.12447026230720
H	0.65297283666797	-2.14243200192819	-1.77957093451755
H	3.08576737771675	-1.87261337686507	-2.21691412595190

TS2

34

C	5.39934040864732	0.79747637461931	-0.71333463372548
O	4.64388264283498	-0.37623025752323	-0.96427720080139
C	3.32637184338069	-0.36120857342249	-0.69051846503048
C	2.64901004523195	0.74343917751444	-0.16346432323226
C	1.27526232517139	0.66714518515715	0.06490493281116
C	0.55988815347455	-0.50459569506241	-0.20762710719521
C	-0.85463041260342	-0.60296465500872	0.16898535341834
C	-1.82908249598947	0.53382558044734	0.43738766151535
C	-1.81589043197375	1.83535117856652	-0.02722412409959
O	-0.92171545913204	2.48243905030586	-0.62738150801242
C	-3.13165323100865	2.61813225939278	0.22426063951841
F	-4.10208631054841	2.21818150533377	-0.62603772637042
F	-2.96946068282824	3.92534876672310	0.02874857622236
F	-3.63178939842488	2.47213093367914	1.46191840112279
C	-3.09276185402343	-0.31813047161623	0.67647585778640
C	-2.91099691689094	-0.86063200928173	-0.70260676276586
C	-1.84125660861204	-1.75609920068071	-0.70373799076955
C	-1.40142644572472	-1.85040011793325	0.75413358728292
C	-2.61971469017748	-1.46159609682085	1.58030161233975
C	1.26300304293595	-1.61846014479177	-0.71169060703840
C	2.61892978379238	-1.54677095323153	-0.96299776561937
H	6.42556124060029	0.57035168402889	-1.00831434801428
H	5.36701167070281	1.05729524565915	0.35399223142370
H	5.01698060135780	1.63761356392783	-1.30965680346255
H	3.17476954528025	1.66637734353568	0.06791367098330
H	0.75254646465347	1.53444994092232	0.45669965162882
H	-4.02776664943835	0.19171827645074	0.89847634109899
H	-3.42567563773348	-0.47624156512471	-1.58408749637092
H	-1.39267611222390	-2.23771991797097	-1.56473455563778
H	-0.71455129066553	-2.64397663565556	1.03974704953308
H	-2.34546783174903	-1.10622741669312	2.57935272779403

H	-3.34698379214630	-2.27855588225749	1.64964435555470
H	0.74242471667907	-2.55225920910674	-0.92207683454492
H	3.16061376715116	-2.39922726408252	-1.37081439734321

NBD3

C	5.20015315625964	0.26207722341317	0.63615278512494
O	4.36653181492391	-0.09200433190214	-0.45459130117306
C	3.03151706181432	-0.08694362597644	-0.27109501035296
C	2.24606119260653	-0.44944880423254	-1.37651598104407
C	0.86571170851333	-0.47650119692275	-1.26930502904346
C	0.22126162282226	-0.12908182215409	-0.06527174092213
C	-1.23640753108986	-0.14478574426795	0.00974876609687
C	-2.08514458059237	0.52745635631497	0.82727711119903
C	-1.76528311922889	1.50195844063651	1.80765463205469
N	-1.53455880736919	2.30461932798791	2.61065921314591
C	-3.52603224343188	0.18731584698339	0.39277631198081
C	-3.70154606527800	0.77447867925130	-1.01703595979260
C	-2.87056405364235	0.10721000399981	-1.82330328245596
C	-2.13735624495485	-0.93232043926066	-0.95980217583252
C	-3.28656288864572	-1.29481497370926	0.01608205145502
C	1.02222391295488	0.22403264460708	1.03179963561569
C	2.41065630617246	0.24715300697027	0.94114019277433
H	6.22709461738718	0.19323531884072	0.27204927440665
H	5.05552740702272	-0.43240736414415	1.47556867670932
H	4.99002527941496	1.28896421215872	0.96642282057180
H	2.74291017058508	-0.71033234657335	-2.31027020811207
H	0.27281760667987	-0.75807469574742	-2.13906296517582
H	-4.30058812131272	0.42446267223522	1.12585363550221
H	-4.34710092117745	1.61485608975145	-1.26274150878110
H	-2.67584514437880	0.27548562439040	-2.88029242916282
H	-1.64280264385649	-1.74697781996786	-1.49407052351658
H	-4.14055286784139	-1.75178998540393	-0.49511340294379
H	-2.95966027186837	-1.90851805392475	0.86327302547111
H	0.55968611304458	0.46491895387966	1.98793110583938
H	2.99783753446662	0.51583680276671	1.81593228036120

QC3

30

C	5.00612012876627	-0.99656434394474	0.50564454746782
O	4.33009548277377	-0.03296797723228	-0.28006993965598
C	2.97673979512078	0.00132345398090	-0.22417449423609
C	2.34247995071389	0.96653882400953	-1.02009933868201
C	0.95920928233529	1.07679273303037	-1.01482365955832
C	0.17009561139985	0.22122728291777	-0.23039755837903
C	-1.30422452966684	0.31281072600386	-0.25183479955064
C	-2.22280334821176	1.35007688307331	-0.84985453798616
C	-3.15481128616775	1.25065236132483	0.37234092821257
C	-3.60331913350399	-0.17764388341589	0.41353634510540
C	-3.41262306736702	-0.83573097625524	-0.93616870108121
C	-2.20986667681687	-0.04903665985161	-1.41224384813908
C	-2.24095094413164	0.19197693381867	0.97851956406792
C	-1.84799090307758	0.06226010522450	2.34091617783823
N	-1.51434006764754	-0.03674521651186	3.44428531624910
C	0.81346106960744	-0.73791846090013	0.55587275378093
C	2.20546377004791	-0.85264600951260	0.57136956566248
H	6.07244457088700	-0.85743430121470	0.31534456580892
H	4.70744172019137	-2.01440509126038	0.21606621320369
H	4.79742893435744	-0.84421840686063	1.57436441249642
H	2.95639865051991	1.62923488700070	-1.62961151143103
H	0.47991576844789	1.84242697262190	-1.62570923431187
H	-1.96390508349203	2.25047036367204	-1.40005576131620
H	-3.62971496145505	2.06693238293714	0.90833480971821
H	-4.38270273817053	-0.47236442121051	1.11292427674436
H	-4.28876417088540	-0.69022037716855	-1.58141930143562
H	-3.20037598522627	-1.90868875724572	-0.84661413326842
H	-1.73558044980023	-0.22444483576121	-2.37582610399076
H	0.22260792839021	-1.41839272334564	1.17060305077739
H	2.67207068206147	-1.60854146792384	1.19881039588900

TS3

C	5.03415472813986	-0.71172748726162	0.59624991310158
O	4.26518485042189	-0.04779161998779	-0.39353607775061
C	2.92475886850528	-0.06586042652499	-0.28318863866659
C	2.20821075603817	0.60687818739651	-1.29232189625603
C	0.82918364928715	0.63595386612264	-1.26130775127822
C	0.11402666374909	0.00846015760349	-0.21902882123096

C	-1.34957579385695	-0.02431044976611	-0.21104102698374
C	-2.14305040268617	1.45218785230207	-0.68789640254334
C	-3.02517505381690	1.27827427295235	0.38523307143871
C	-3.57425757836511	-0.10444558703743	0.37042559889648
C	-3.54003573727384	-0.48922220203505	-1.11351353330603
C	-2.17729886882693	0.12044010136029	-1.43014546359507
C	-2.21216875960566	-0.62061825169417	0.89742431719734
C	-1.90298591964039	-0.47343808149476	2.25310935401035
N	-1.65909086792383	-0.43151209979826	3.39849745160979
C	0.83854164127937	-0.64890595385718	0.78215438508533
C	2.23271816573845	-0.69917311936546	0.75413070990828
H	6.07991374239071	-0.57430471556926	0.31494471597493
H	4.79121151343750	-1.78315267716197	0.61902512079012
H	4.85273333537984	-0.27061885205837	1.58623221691917
H	2.76393884038962	1.09617485229678	-2.09127657344560
H	0.29604800570992	1.16127195251675	-2.05364326757837
H	-1.50215971491236	2.30391663858406	-0.88138149377673
H	-3.15734041051738	1.97649406395630	1.21314418710127
H	-4.45763752609420	-0.31588368343511	0.97193226407482
H	-4.33949673446990	-0.01681474383168	-1.69612646576896
H	-3.55451938599997	-1.57520055357321	-1.25261125001398
H	-1.71489573707173	0.05473006489706	-2.41254706534573
H	0.31656074546100	-1.14958149369569	1.59420776981015
H	2.76249298513349	-1.22746001184017	1.54284465162161

NBD4

C	4.60998495123549	1.35150620737092	2.02411840689310
C	4.57024175154875	1.66525945708706	0.54174745976987
O	3.86832268950157	0.64609235251418	-0.18499157725165
C	2.53033144347559	0.70602979487860	-0.20412628692084
O	1.90914981860056	1.59401779943764	0.34262274964383
C	1.95795037050631	-0.43020739902965	-0.94255614352806
C	0.66373699947025	-0.82387169870751	-1.03135373157175
C	-0.59247107770929	-0.25158706659092	-0.53895944132115
C	-1.58991150828563	-1.11190661816239	-0.05533314391614
C	-2.80986181405747	-0.63578164324358	0.41655599926855
C	-3.07338306691099	0.74182887706913	0.38278186059828
N	-4.26814857210748	1.32229983740101	0.83116881068925

C	-5.42253911143986	0.71010214106523	1.23344783882926
C	-6.50821710700652	1.66875380166308	1.66004001910579
O	-5.56944126755351	-0.50246991283530	1.25241259133104
C	-2.09164416037419	1.61095869962159	-0.12559645889443
C	-0.87265971388361	1.12505482840529	-0.57144801467985
C	0.68672534050648	-2.19990950494278	-1.72987788837900
C	1.39145457787578	-3.17279099872301	-0.77044166875852
C	2.66585277529310	-2.77965079642040	-0.68322304856739
C	2.81420202594315	-1.53919281868111	-1.58116843867972
C	1.84930448059529	-1.94496135008397	-2.72036539088456
H	5.03623774948977	0.35421640550050	2.18856524677193
H	5.23998626934762	2.08922393639044	2.53600546703163
H	3.60392178538007	1.38848062974588	2.45769069320496
H	5.57578590215188	1.67161232558136	0.11014003246814
H	4.08956965719803	2.63078725814770	0.34772163362016
H	-1.40523765822653	-2.18618140075884	-0.02939414948570
H	-3.55184244444640	-1.32526486941888	0.80138777631186
H	-4.28517888531374	2.33555019188785	0.82664447109606
H	-7.39738402413680	1.09943233649649	1.93953372416405
H	-6.74714328807486	2.35915861620672	0.84075083769440
H	-6.16312949975124	2.26573371863671	2.51435861149459
H	-2.29883327069712	2.68079306581324	-0.17122932162756
H	-0.12879560386396	1.81369002979181	-0.96370441327825
H	-0.27748376252602	-2.52290343211635	-2.13143250185036
H	0.89381077379994	-3.97958003651089	-0.23628531593504
H	3.45617808023154	-3.19497558326712	-0.06144793697476
H	3.83306183479714	-1.24712710669300	-1.84080493111604
H	2.15993535081971	-2.85267959892611	-3.24920529973286
H	1.64762120859725	-1.12957047560064	-3.42476912663310

QC4

C	-4.21114569516667	3.26957679878877	0.30453162900275
C	-4.23043247588503	2.14738131563615	1.32321456090904
O	-3.81803250541364	0.90243039799408	0.73803308827871
C	-2.50432575040732	0.68541557631009	0.60437789100668
O	-1.66430338268657	1.48114896798138	0.96728738218208
C	-2.23292455273976	-0.59969576996204	-0.05111358724320
C	-2.89255229118533	-1.05777686247666	-1.35134243210181

C	-1.55535596180443	-1.66478524004363	-1.80930325603779
C	-1.23172587251113	-2.67759055868865	-0.74498615787133
C	-2.47300965308190	-3.06358359581860	0.02870034609130
C	-3.22627035418094	-1.75573433069922	-0.07624787224144
C	-0.89172853536995	-1.21123935241512	-0.52631907816229
C	0.49345516096067	-0.73448440294948	-0.32007383224204
C	1.44970065842016	-0.88347718549265	-1.32981625577248
C	2.78088930310097	-0.50957917554679	-1.14806559413738
C	3.18414912053399	0.04554081642489	0.07390168983599
N	4.49326084757965	0.48267108093254	0.34753254017830
C	5.60698719685450	0.39837192444737	-0.43458926881314
C	6.83327642234014	1.04090846791649	0.17032659266838
O	5.62465959371122	-0.14865821473451	-1.52870971169714
C	2.23180074612309	0.19913402026465	1.09439249399219
C	0.91276247973108	-0.19030997598794	0.90271149001844
H	-3.18374080642433	3.52337563617142	0.01935029171244
H	-4.77192315819717	2.97559346532061	-0.59142569824094
H	-4.68564980777523	4.15972949031845	0.73531560057296
H	-3.57803268212785	2.36649230042879	2.17607851851957
H	-5.24479779164167	1.95926712158922	1.68766457654621
H	-3.58730536089669	-0.47391664505134	-1.94802542968044
H	-1.14998403850948	-1.66235797719034	-2.81746120808902
H	-0.39875301043053	-3.36041669581205	-0.90010947980573
H	-2.24993397975921	-3.33037010276384	1.06981419520901
H	-3.02124687747211	-3.88719320698454	-0.44750876971359
H	-4.19578679982563	-1.59199067508186	0.38684092837494
H	1.15268518154394	-1.30014053742580	-2.29265508927252
H	3.49644470200747	-0.63783133751615	-1.95171368275699
H	4.61789148024930	0.93610315344537	1.24500067741732
H	7.67663347052133	0.91416788418279	-0.51200133194682
H	6.64971897281837	2.10955822934186	0.34180613478088
H	7.06390532140793	0.57932010367298	1.13926944194222
H	2.53732696923153	0.62887165351163	2.04963069022786
H	0.19338371635722	-0.06390656203830	1.70938697635883

TS4

C	-4.43123660185970	2.80658582518667	0.13579885259091
C	-4.14482632326914	2.11907700069005	1.45889114388291

O	-3.69161550835276	0.78468517641632	1.26054023422895
C	-2.36728078415336	0.60947948031599	0.91840804198336
O	-1.58757336101938	1.56911322559305	0.98100999134558
C	-2.09291514139375	-0.72957284706430	0.56427078325357
C	-2.80733372824737	-0.91396923796817	-1.41445852534708
C	-1.59642745212851	-1.42148919602971	-1.89270121354385
C	-1.21242358743670	-2.49621946027008	-0.87870167395706
C	-2.52620232421242	-2.97228416347293	-0.26918016537172
C	-3.17153917088861	-1.58954664025898	-0.13762572116406
C	-0.91122634877794	-1.18307256376424	-0.26082362489736
C	0.46927616455115	-0.69239662447003	-0.17969398147132
C	1.42170036606211	-0.99510296412285	-1.16522069218140
C	2.75253431306782	-0.62284143937016	-1.03253888858166
C	3.17185603961435	0.05224026554783	0.12723447604839
N	4.49036888790910	0.46166084924520	0.36030908970772
C	5.57566487557093	0.37209350002320	-0.46573258515901
C	6.85262397713611	0.94973413464641	0.09453303891589
O	5.53340109108367	-0.12535995894014	-1.58098725222522
C	2.22928100711053	0.34088331058176	1.12716526715386
C	0.89664437560544	-0.01224979892364	0.97191305093032
H	-3.51458791899083	2.88225727834062	-0.46214478202940
H	-5.18078009814143	2.24000998875337	-0.43167556800945
H	-4.81821116745599	3.81822997679435	0.31213220584449
H	-3.40400647230092	2.67910167174527	2.04106770360391
H	-5.06016595345761	2.02649879177113	2.05464873845123
H	-3.32822831927375	-0.03916155500777	-1.80739926068942
H	-1.01064890407577	-1.05096223513585	-2.72505268385317
H	-0.39195320410664	-3.17044599038029	-1.11601161076411
H	-2.38121357821068	-3.45009495483477	0.70575018034466
H	-3.07174553065688	-3.64011024587194	-0.94606549920316
H	-4.19429149676383	-1.49352349992725	0.21986070343870
H	1.12539160026402	-1.52760816535446	-2.06849016865240
H	3.46325900918602	-0.85602448706917	-1.81608905425728
H	4.65155244573368	0.90517802093017	1.25615345059825
H	7.64196963364021	0.19552663182179	-0.00123586850937
H	7.13339160630394	1.81392129479760	-0.52058190677352
H	6.76092278123002	1.26096039750868	1.13959910542819

H	2.55105128056059	0.85881370799307	2.03140275573112
H	0.17550352054430	0.23600549953418	1.74542191315904

References

1 J. L. Elholm, A. E. Hillers-Bendtsen, H. Hölzel, K. Moth-Poulsen and K. V. Mikkelsen, *Phys. Chem. Chem. Phys.*, 2022, **24**, 28956-28964.
2 A. E. Hillers-Bendtsen, J. L. Elholm, O. B. Obel, H. Hölzel, K. Moth-Poulsen and K. V. Mikkelsen, *Angewandte Chemie International Edition*, 2023, e202309543.
3 G. Landrum, *Greg Landrum*, 2013, **8**, 31.
4 C. Bannwarth, S. Ehlert and S. Grimme, *Journal of chemical theory and computation*, 2019, **15**, 1652-1671.
5 Y. Zhao and D. G. Truhlar, *Theoretical chemistry accounts*, 2008, **120**, 215-241.
6 F. Weigend and R. Ahlrichs, *Physical Chemistry Chemical Physics*, 2005, **7**, 3297-3305.
7 T. Yanai, D. P. Tew and N. C. Handy, *Chemical physics letters*, 2004, **393**, 51-57.
8 S. Smidstrup, A. Pedersen, K. Stokbro and H. Jónsson, *The Journal of chemical physics*, 2014, **140**.
9 F. Neese, *Wiley Interdisciplinary Reviews: Computational Molecular Science*, 2022, **12**, e1606.
10 V. Barone and M. Cossi, *The Journal of Physical Chemistry A*, 1998, **102**, 1995-2001.
11 Z. Wang, J. Udmark, K. Boerjesson, R. Rodrigues, A. Roffey, M. Abrahamsson, M. B. Nielsen and K. Moth-Poulsen, *ChemSusChem*, 2017, **10**, 3049-3055.
12 Z. Wang, A. Roffey, R. Losantos, A. Lennartson, M. Jevric, A. U. Petersen, M. Quant, A. Dreos, X. Wen, D. Sampedro, K. Borjesson and K. Moth-Poulsen, *Energy Environ. Sci.*, 2019, **12**, 187-193.
13 W. Alex, P. Lorenz, C. Henkel, T. Clark, A. Hirsch and D. M. Guldi, *J. Am. Chem. Soc.*, 2022, **144**, 153-162.
14 P. Lorenz and A. Hirsch, *Chem. Eur. J.*, 2020, **26**, 5220-5230.
15 B. E. Tebikachew, F. Edhborg, N. Kann, B. Albinsson and K. Moth-Poulsen, *Phys. Chem. Chem. Phys.*, 2018, **20**, 23195-23201.
16 H. Kamogawa and M. Yamada, *Macromolecules*, 1988, **21**, 918-923.
17 A. Tsubata, T. Uchiyama, A. Kameyama and T. Nishikubo, *Macromolecules*, 1997, **30**, 5649-5654.
18 K. Stranius and K. Börjesson, *Sci. Rep.*, 2017, **7**, 41145.
19 C. G. Hatchard, C. A. Parker and E. J. Bowen, *Proc. R. Soc. A*, 1956, **235**, 518-536.
